# Receptor-mediated endocytosis 8 (RME-8)/DNAJC13 is a novel positive modulator of autophagy and stabilizes cellular protein homeostasis

**DOI:** 10.1007/s00018-020-03521-y

**Published:** 2020-04-22

**Authors:** Anna S. Besemer, Joanna Maus, Mirjam D. A. Ax, Anna Stein, Stella Vo, Christian Freese, Karsten Nalbach, Christian von Hilchen, Ines F. Pfalzgraf, Ingrid Koziollek-Drechsler, Beate Silva, Heike Huesmann, Fatima Boukhallouk, Luise Florin, Andreas Kern, Christian Behl, Albrecht M. Clement

**Affiliations:** 1 Institute of Pathobiochemistry, University Medical Center of the Johannes Gutenberg-University, Duesbergweg 6, 55128 Mainz, Germany; 2 Institute of Medical Microbiology and Hygiene, University Medical Center of the Johannes Gutenberg-University, 55101 Mainz, Germany; 3 Institute for Virology and Research Center for Immunotherapy (FZI), University Medical Center of the Johannes Gutenberg-University, 55101 Mainz, Germany

**Keywords:** Proteostasis, RME-8, DNAJC13, Autophagy, ATG9A, Recycling endosome, *C. elegans*

## Abstract

The cellular protein homeostasis (proteostasis) network responds effectively to insults. In a functional screen in *C. elegans*, we recently identified the gene *receptor-mediated endocytosis 8* (*rme-8;* human ortholog: *DNAJC13*) as a component of the proteostasis network. Accumulation of aggregation-prone proteins, such as amyloid-β 42 (Aβ), α-synuclein, or mutant Cu/Zn-superoxide dismutase (SOD1), were aggravated upon the knockdown of *rme-8/DNAJC13* in *C. elegans* and in human cell lines, respectively. DNAJC13 is involved in endosomal protein trafficking and associated with the retromer and the WASH complex. As both complexes have been linked to autophagy, we investigated the role of DNAJC13 in this degradative pathway. In knockdown and overexpression experiments, DNAJC13 acts as a positive modulator of autophagy. In contrast, the overexpression of the Parkinson’s disease-associated mutant DNAJC13(N855S) did not enhance autophagy. Reduced DNAJC13 levels affected ATG9A localization at and its transport from the recycling endosome. As a consequence, ATG9A co-localization at LC3B-positive puncta under steady-state and autophagy-induced conditions is impaired. These data demonstrate a novel function of RME-8/DNAJC13 in cellular homeostasis by modulating ATG9A trafficking and autophagy.

**Electronic supplementary material:**

The online version of this article (10.1007/s00018-020-03521-y) contains supplementary material, which is available to authorized users.

## Introduction

A fine-tuned, multi-component molecular network is in place to preserve protein homeostasis (proteostasis) on the cellular level, particularly under acute and chronic challenges. Despite the flexibility of the system, the ability to adapt to stressors generally declines with age [1, 2]. As a consequence, aged cells are prone for the accumulation of misfolded proteins which, in turn, exacerbates the imbalance of the proteostasis network. This vicious cycle, resulting in the accumulation of protein aggregates, is at least in part the molecular basis for the development of several age-related neurodegenerative diseases, like Parkinson’s disease and amyotrophic lateral sclerosis [3].

Macroautophagy (hereafter termed autophagy) is one major pathway to remove misfolded proteins and its activity is reduced with age on the cellular and the organism levels [4]. Generally, autophagy directs cytosolic cargos, but also invading pathogens, to lysosomal degradation [5] and its dysfunction has been linked to severe pathologic conditions including neurodegenerative diseases [6]. The degradative pathway can be induced by starvation conditions and the treatment with compounds such as rapamycin that inhibit the Ser/Thr kinase *mechanistic target of rapamycin* (mTOR) [7]. The inhibition of mTOR results in a translocation of the ULK complex from the cytosol to the ER. The detailed mechanism of phagophore initiation and elongation is still enigmatic [8]. The processes depend on the ubiquitin-like ligation complex ATG12-ATG5/ATG16L1 which is responsible for the lipidation of MAP1LC3 (shortly LC3), a member of the Atg8 protein family. Membrane-anchored LC3-II is essential for phagophore elongation, and acts also as an attachment site for autophagy cargo receptors such as SQSTM1/p62 [9]. Membranes and lipids are delivered from different sources within cells such as the endoplasmic reticulum [10], mitochondria [11, 12], the Golgi–endosomal system [13], or lipid droplets [14, 15], indicating that lipid transport processes that are supported by RAB GTPases are essential for the maturation of phagophores to autophagosomes [16]. An important component for the maturation of autophagosomes and the delivery of lipids towards the growing phagophore is ATG9A [17, 18]. Several lines of evidence suggested that the routing of ATG9A through the recycling endosome is an essential step for proper autophagosome biogenesis [19–24].

*Rme-8* was initially identified as a component of the endocytosis machinery in *C. elegans* [25], belongs to the DNAJ/HSP40 protein family, and is highly conserved [25–28]. The human ortholog of *rme-8* is *DNAJC13*, a large protein containing 2243 amino acids. Due to the presence of the central DNAJ domain, DNAJC13 binds HSC70 (heat shock cognate 70) [27, 29] and acts as a co-chaperone in mediating HSC70 cellular functions. Within the cell, DNAJC13 is primarily localized at membranous structures. This interaction is mediated, at least in part, by the binding to phosphoinositides (PI) through its N-terminal sequence [30]. RME-8/DNAJC13 is involved in several endosomal functions such as protein sorting [26, 31], endosomal tubulation [32], transport processes including the retrograde transport from the endosome to the trans-Golgi network (TGN) [27, 33, 34], the formation of endosomal degradative microdomains [35], and the recycling of membrane receptors [36]. In addition, it has been proposed that DNAJC13 might act as a scaffold to organize the retromer and WASH complexes, since DNAJC13 interacts with FAM21 [32], a component of the WASH complex, and SNX1 [33, 34], a constituent of the sorting nexin dimer tightly linked to the core retromer trimer, consisting of VPS26, VPS35, and VPS29.

There is growing evidence that retromer dysfunction is linked to neurodegeneration [37, 38]. More specifically, point mutations within *DNAJC13* have been associated with rare familial cases of Parkinson’s disease (PD) with Lewy body pathology [39]. The expression of mutant DNAJC13 resulted in the accumulation of α-synuclein in mammalian cells, and in Drosophila [40, 41], pointing towards a role of DNAJC13 in proteostasis. It is of note that the DNAJC protein family as a subclass of HSP40 heat shock proteins received attention due to the identification of various mutations that are linked to PD and other age-related neurological disorders [42].

In a previous screen, we uncovered that *rme-8*/*DNAJC13* is involved in maintaining proteostasis in acute heat stress models in *C. elegans* [43, 44], and here, we transferred these findings in human cell lines. The accumulation of aggregation-prone proteins upon *rme-8*/*DNAJC13* knockdown and its functional association with the retromer and WASH complexes, both of which prompted us to investigate the role of RME-8/DNAJC13 in autophagy. We could demonstrate that RME-8/DNAJC13, but not the PD-associated mutant DNAJC13(N855S), is a positive modulator of this degradative process. Moreover, we found that DNAJC13 impacts on the subcellular distribution of ATG9A. Since ATG9A localization at and its transport from the recycling endosome was reduced at DNAJC13 knockdown conditions, we suggest that DNAJC13 modulates autophagy by affecting ATG9A trafficking.

## Materials and methods

### *C. elegans*

*Caenorhabditis elegans* strains were obtained from the *Caenorhabditis* Genetics Center (CGC), which is funded by NIH Office of Research Infrastructure Programs (P40 OD010440). Strains were cultivated on HB101 *Escherichia coli* containing nematode growth medium (NGM) plates according to the standard procedures. The large-scale, unbiased RNAi screen of chromosome one in nematodes expressing a luciferase-GFP fusion protein (Luc-GFP) in the body wall muscle cells [45] was performed as described [44]. To analyze the effect of the newly identified gene *rme-8* on the paralysis of human Aβ42-expressing *C. elegans*, synchronized CL2006 (dvIs2 [pCL12(P_unc-54_-hAβ)/pRF4]) nematodes [46] were cultivated at 15 °C and placed on RNAi plates at the L4 stage. Worms were transferred onto new plates daily and paralysis was tested by gently tipping the nose of the nematode with a platinum wire. Worms that moved their nose but were not able to move their body were scored as paralyzed. Nematodes showing additional aberrant phenotypes were not included into the statistics. The staining of amyloid structures by thioflavin was performed as previously described [47].

The efficiency of the *rme-8* knockdown was monitored by quantitative real-time PCR (qPCR). For RNA extraction and quantification, RNA was isolated from total lysate of 15–20 worms using Absolutely RNA Nanoprep Kit (Agilent, 400753). Reverse transcription and qPCR were performed as described in detail in the Supplemental Method section.

### Cell culture

Human embryonic kidney cells 293 (HEK293A or HEK293T) were cultivated in high glucose containing Dulbecco’s modified Eagle’s medium (DMEM, Life Technologies, 41965062) supplemented with 10% fetal calf serum (FCS) (PAA Laboratories, A15-101), 1% sodium pyruvate (Life Technologies, 1136-088), and a mixture of antibiotics and antimycotics (Life Technologies, 15240-112). HeLa cells were cultivated in DMEM supplemented with 10% FCS, 1% Glutamax (Life Technologies, 35050061), 1% modified Eagle’s medium nonessential amino acids (Life Technologies, 11140050), and antibiotics (Life Technologies, 15140122). Cultures were kept at 37 °C in a humidified atmosphere containing 5% CO_2_.

For transient expression, HEK293 cells were transfected by calcium phosphate precipitation as described earlier [48] and specified in the supplemental material. A set of two siRNAs against *DNAJC13* or *nonsense* siRNA (Suppl. Table 1) (MWG Eurofines Genomics, Sigma) were introduced by electroporation using the Nucleofactor 2B system (Lonza). 48 h after transfection cells were exposed to 4 µM bafilomycin A_1_ (LC Laboratories, B-1080) and/or 10 µM rapamycin (Enzo, BML-A275-0025) for 4 h. Stock solutions were prepared in DMSO (Roth, A994.2). HeLa cells were transfected with siRNA against *DNAJC13* or *nonsense* siRNA and GFP-LC3 and mCherry-ATG9A expressing plasmids (kind gift from I. Dikic, Frankfurt, Germany) by electroporation and plated onto glass cover slips. Cells were cultivated for 48 h and then treated with DMSO (control) or rapamycin (10 µM) for 4 h.

### Generation of stable Luc-GFP expressing HEK293A cells and heat stress experiments

A luciferase–GFP fusion construct [45] was subcloned in the pLHCX vector (Invitrogen) (pLHCX:Luc-GFP). Viruses were produced as previously described [49] and specified in the Supplemental Methods. Virus-containing supernatant was incubated with HEK293A cells for 36 h, followed by an incubation of transfected cells in complete medium containing 250 µg/ml hygromycin (Life Technologies, 10687010) to select for stably transfected cells.

For heat stress experiments, stably transfected Luc-GFP cells were cultivated in six-well plates after transient transfection of siRNA by electroporation. 48 h after transfection, the medium was changed and plates were placed for 1 h in a water bath inside an incubator with a temperature of 41 °C. The heat stress paradigm was established as such that the stress did not alter cellular morphology and induced luciferase accumulations in about 5% of cells after *nonsense* siRNA transfection. The GFP signal was documented by a conventional Zeiss inverted microscope equipped with a monochrome camera (Spot RT, National Diagnostics). At least 200 cells per experiment and condition were analyzed, and the number of cells with bright fluorescent Luc-GFP accumulations was determined. Numbers were normalized and statistics were performed relative to the number of cells with accumulations under *nonsense* siRNA conditions.

### HPV pseudovirion transduction

HeLa cells were transfected with siRNA against *DNAJC13* or *nonsense* siRNA using Lipofectamine RNAiMAX (Invitrogen, 13778150) according to the manufacturer’s instructions. 24 h post-transfection, GFP-LC3B, and mCherry-ATG9A expressing plasmids were introduced by Lipofectamine (Invitrogen, 11668019) for additional 24 h. Afterwards, human papillomavirus type 16 (HPV16) pseudovirions were added. HPV pseudovirions were prepared as described earlier [50]. Briefly, HEK293TT (SV40 large T antigen expressing HEK293T cells) cells were co-transfected with expression plasmids for HPV16 L1 and L2 as well as with pcDNA3.1/Luciferase plasmids [51] and lysed 48 h post-transfection. Pseudovirions were purified by an Optiprep (Sigma, D1556) gradient centrifugation. The addition of 100 luciferase vector-positive pseudovirions per cell resulted in labeling of all HeLa cells with HPV virions. Transduction/infection efficiencies of HPV pseudovirions were assessed by quantification of luciferase expression 24 h post-infection. Cells were lysed with cell culture lysis reagent and relative luciferase activity was measured with the luciferase assay system (Promega, E4030) according to the manufacturer’s instructions, using the Tristar LB 941 luminometer (Berthold Technologies). Luciferase activities were normalized to lactate-dehydrogenase (LDH) activity for cell viability (CytoTox-One, Promega, G7891) and are depicted as percentages of infection relative to the luciferase activity in control-treated cells.

### Generation of expression constructs

For the generation of C-terminally tagged DNAJC13 constructs, FLAG and EGFP were amplified with forward and reverse primers adding a BamHI and an XbaI site, respectively. *DNAJC13* cDNA was excised from a DNAJC13 expression plasmid [26] (kind gift from K. Sekiguchi, Osaka, Japan) and fragments were re-ligated in the pEF-BOS-EX vector (kind gift of S. Nagata, Kyoto, Japan) (pEFBos:DNAJC13-FLAG; pEFBos:DNAJC13-EGFP). DNAJC13 mutations were introduced in the pEF-BOS-DNAJC13 Vectors with the QuikChange Lightning Site-directed Mutagenesis kit (Agilent, 210518). DNAJC13(N855S): forward (5′-gcgatgataaagctcactgaaaaattcatacgatctcttaattgatcc-3′) and reverse primers (5′-ggatcaattaagagatcgtatgaatttttcagtgagctttatcatcgc-3′); DNAJC13(W20A): forward (5′-cgcttatacttccccctcgctgaatgttttgttgtgtagaaacatcg-3′) and reverse primers (5′-gcatgtttctacacaacaaaacattcagcgagggggaagtataagcg-3′). Wild-type α-synuclein was amplified from a prion-promotor-driven expression plasmid [52] (kind gift from G. Auburger, Frankfurt, Germany) and introduced in the pEGFP-N1 vector. SOD1(G85R) was expressed within the pEGFP-N1 vector as described earlier [53]. The sequence of all constructs was verified by sequencing analysis (MWG). N-terminally tagged GFP-RAB11 [54] (generously provided by M. I. Colombo, Mendoza, Argentina, via R. Prange, Mainz, Germany) and GFP-RAB7 (kind gift from A. Helenius, Zurich, Switzerland, via M. Husmann, Mainz, Germany) were expressed from pEGFP-C plasmids.

### Immunocytochemistry and microscopy

Cells were grown on glass coverslips and treated as described above. Cells were fixed with 4% paraformaldehyde (Sigma, P6148) and, if necessary, permeabilized with − 20 °C-cold methanol (95%) for 6 min, washed with phosphate-buffered saline (PBS) (Sigma, D8537) and incubated with primary antibodies specifically detecting LC3B (Nano Tools, 0260-100), sequestosome 1/p62 (SQSTM1) (Progen, GP62-C), early endosome antigen 1 (EEA1) (BD Transduction Labs, 616457), ATG9A (Abcam, ab108338), cation-independent mannose-6-phosphate receptor (CI-M6PR) (Abcam, ab8093), lysosomal-associated membrane protein 2 (LAMP2) (Abcam, ab25631), trans-Golgi network protein 2 (TGN46) (ABD Serotec, AHP500), ATG16L1 (MBL, PM04), and FLAG (Sigma, F1804). Subsequently, primary antibodies were detected with species-specific secondary antibodies coupled to fluorescence dyes. After another washing step with PBS, DAPI (Calbiochem, 382061) or Hoechst 33342 (Sigma, B2261) was added for nuclear counterstaining. Coverslips were mounted and analyzed with Leica SPE or SP5 confocal microscopes (at the Institute for Molecular Biology, Mainz, Germany) or a Zeiss 710 confocal microscope. Images were carefully taken avoiding overexposure within cells. In case of GFP-RAB11 and GFP-RAB7 transfected cells, only cells with a dotted distribution of GFP were chosen for analysis and the GFP signal was monitored. We intentionally analyzed unprocessed confocal single slice images using the plot profile tool within the FIJI software [55]. Lines were drawn throughout cells based on the distribution of ATG9A blinded to the GFP signals (Suppl. Figure 7C). At least 13 lines resulting in about 85 puncta per cell and between 9 and 37 cells per condition were analyzed. Subsequently, intensity profiles were compared with peak intensities from co-stainings on the very same lines, and overlapping peaks were counted and normalized to all ATG9A-positive signals. Fluorescence imaging for virus experiments was performed using a Zeiss Axiovert 200 M microscope equipped with a Plan-Apochromat 100 × (1.4 NA). About eight randomly selected and unprocessed images per condition in three independent experiments (about 100 cells in total) were analyzed using Axiovision co-localization software 4.7 (Zeiss, Jena, Germany). A fixed threshold was set to remove background staining for each experiment.

### Protein extraction, fractionation, and Western blotting

Total cell extracts were prepared in lysis buffer containing 62.5 mM Tris–HCl, 2% (w/v) SDS and 10% sucrose (pH 8) supplemented with EDTA-free protease (Roche, 04693159001) and phosphatase inhibitor cocktails (Roche, 04906837001) and sonicated four times at 60 Hz for 10 s on ice. Fractionation experiments after overexpression of aggregation-prone proteins were performed as described earlier [56] and specified in the Supplemental Methods. For Western blotting, equal amounts of proteins were separated by SDS-PAGE under denaturing conditions. Proteins were transferred on nitrocellulose membrane and proteins were detected by specific antibodies recognizing SQSTM1 (Progen, GP62-C), LC3B (Novus, NB-100-2220), FLAG (Sigma, F1804), tubulin (Sigma, T9026), SOD1 (Epitomics, 2018-1), ATG9A (Abcam, ab108338), histone H3 (Abcam, ab47915), α-synuclein (Abcam, ab27766), and DNAJC13 (kind gift from M. Farrer, Vancouver, and P. McPherson, Montreal, Canada). Species-specific secondary antibodies coupled to horseradish peroxidase (Dianova) were used to develop the Western blot with the Fusion system (Peqlab) or the Amersham Imager 600 (GE). Densitometry was performed with the AIDA software (Raytest) or ImageJ.

### Statistical analysis

For the comparison of two groups, variance was determined and Student’s *t* test (two-tailed, unpaired) was applied accordingly. In some cases, Mann–Whitney *U* test or the one-way ANOVA with Bonferroni or Games–Howell correction for multiple comparisons was used (SPSS software, IBM; SigmaStat, Systat; Prism). Statistical analysis for virus experiments was performed using Statistical Software R (2017, version 3.3.1) from R Core Team (R: A language and environment for statistical computing. R Foundation for Statistical Computing, Vienna, Austria). All values were tested for normality using the Shapiro–Wilk test. As the values were not normally distributed, Wilcoxon rank-sum test was applied.

## Results

### *rme-8*/DNAJC13 knockdown affects proteostasis in *C. elegans* and HEK293 cells

**Fig. 1:**
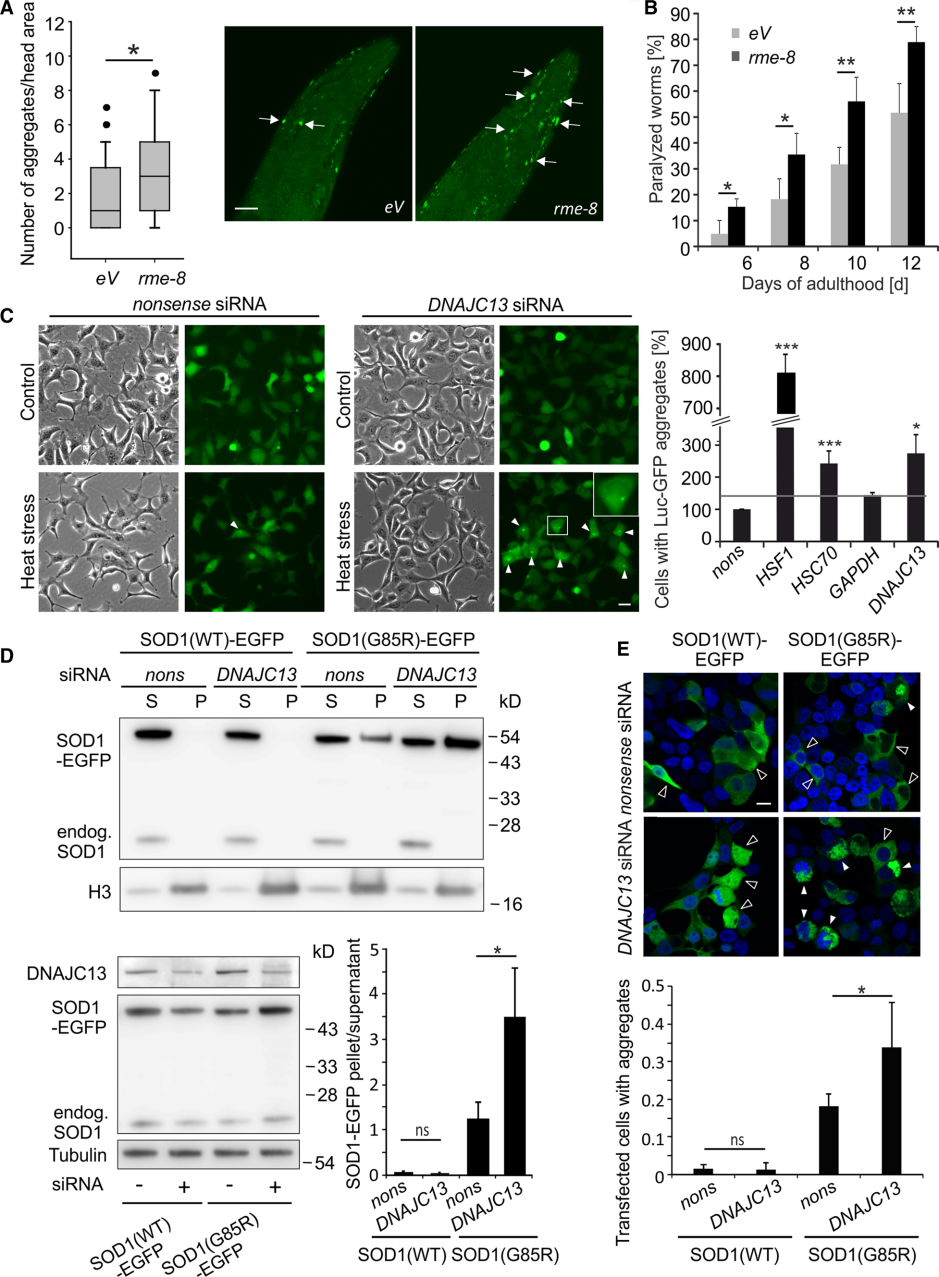
Knockdown of *rme-8*/*DNAJC13* affects proteostasis. **a**, **b** *C. elegans* expressing Aβ42 in body wall muscle cells (CL2006) were fed with *rme-8* RNAi or bacteria containing empty vector (eV) starting from the L4 stage. **a** Two-day adult animals show an increased number of thioflavin-positive accumulations (arrows) in the head region under *rme-8* knockdown conditions. About 40 worms were counted and results were represented in a box-plot (*n* = 41/42; Mann–Whitney *U* test: *p ≤ 0.05). Micrographs represent maximum intensity projections of the head region of worms (scale bar: 20 µm). **b** CL2006 under *rme-8* knockdown conditions developed an aggravated paralysis compared to control nematodes (eV). In total, 151 (eV) and 202 (*rme-8*) worms were analyzed (*n* = 4; mean ± SEM; *t* test: *p ≤ 0.05, **p ≤ 0.01). **c** Phase-contrast and epi-fluorescent pictures of HEK293A stably expressing Luc-GFP under control conditions or after heat stress and siRNA treatment. At least 200 cells per experiment and condition were analyzed and the number of cells with bright fluorescent Luc-GFP accumulations (arrowheads) was determined. The inset shows an enlargement of the boxed cell. Numbers were normalized and statistics were performed relative to the number of cells with accumulations under *nonsense* siRNA conditions (*n* = 3; mean ± SEM; *t* test: *p ≤ 0.05, ***p ≤ 0.001) (scale bar: 20 µm). **d**, **e** HEK293T cells were transiently transfected with EGFP-tagged wild-type SOD1(WT) or ALS-causing mutant SOD1(G85R) and *nonsense* siRNA or *DNAJC13* siRNA. **d** Cell lysates were separated in a soluble (S) and an aggregate-enriched fraction (P) and the relative distribution of the SOD1-EGFP variants were quantified (*n* = 3; mean ± SD; *t* test: *p ≤ 0.05) (lower right panel). Endogenous SOD1 and histone H3 served as markers for the soluble and the aggregate-enriched fraction, respectively (upper panel). Representative Western blots showing the expression levels of transgenic SOD1-EGFP variants and DNAJC13 levels in total cell lysates (lower left panel). **e** Confocal images of HEK293T cells transfected with SOD1(WT) and SOD1(G85R) and *nonsense* or *DNAJC13* siRNA. Images were taken with the same exposure conditions. Open arrows point to cells without aggregates; full arrows point to aggregates. The number of transfected cells with SOD1(G85R) aggregates was determined (*n* = 4; one-way ANOVA with Bonferroni correction; *p ≤ 0.05) (scale bar: 10 µm)

Previously, we detected the gene *rme-8* in an RNAi screen using *C. elegans* expressing the protein folding sensor luciferase-GFP to identify modulators of proteostasis [43, 44]. To confirm the role of RME-8 in proteostasis, nematodes expressing human amyloid-β 42 (Aβ42) in body wall muscle cells were employed. This transgenic line (CL2006) is characterized by the age-dependent appearance of Aβ42 aggregates and an associated progressive paralysis [46]. Upon *rme-8* RNAi treatment, the number of aggregates was increased compared to controls (eV) as shown by thioflavin staining (Fig. 1a). Consistently, the paralysis phenotype was aggravated in Aβ42-expressing nematodes (Fig. 1b).

DNAJC13 is involved in proteostasis also in human cells. We generated an HEK293A cell line stably expressing luciferase-GFP and analyzed the generation of its aggregates following a heat stress paradigm. Exposure to heat stress under control conditions with *nonsense* siRNA or siRNA directed against *glyceraldehyde-3-phosphate-dehydrogenase* (*GAPDH*) had only a very mild effect on luciferase–GFP inclusion formation and did not induce overt morphological changes in these cells (Fig. 1c). In contrast, the knockdown of *heat shock factor 1* (*HSF1*) and *heat shock cognate 70* (*HSC70*) resulted in a significantly increased number of cells with luciferase-GFP accumulations compared to *nonsense* siRNA and served as positive control (Fig. 1c; Suppl. Figure 1A, B). Under the same conditions, the number of cells with luciferase-GFP inclusions treated with *DNAJC13* siRNA was significantly increased compared to *nonsense* siRNA controls (Fig. 1c).

The relevance of *DNAJC13* for maintaining proteostasis was also shown by the analysis of mutant Cu/Zn-superoxide dismutase (SOD1) and α-synuclein aggregation in human cell lines. Mutant SOD1 variants cause familial forms of amyotrophic lateral sclerosis and α-synuclein is the main constituent of Lewy bodies which are a prominent pathological hallmark of PD. The ectopic expression of SOD1 carrying the point mutation SOD1(G85R) [53, 57] or α-synuclein [58] results in the formation of inclusions. HEK293T cells transiently overexpressing either SOD1(G85R) or α-synuclein were additionally co-transfected with *nonsense* or *DNAJC13* siRNA (Fig. 1d, e; Suppl. Figure 1C, D). The separation of cell lysates in a soluble and an aggregate-enriched fraction revealed that reduced DNAJC13 protein levels resulted in an accumulation of mutant SOD1 and α-synuclein within the aggregate fraction (Fig. 1d; Suppl. Figure 1D). Concomitant with the biochemical data, the number of SOD1(G85R)-transfected cells bearing aggregates is increased under *DNAJC13* knockdown conditions (Fig. 1e), emphasizing that diminished DNAJC13 levels facilitated protein aggregation; *DNAJC13* knockdown did not promote SOD1(WT) aggregation (Fig. 1d, e).

### Decreased DNAJC13 levels reduce autophagic activity under basal and stimulated conditions

**Fig. 2:**
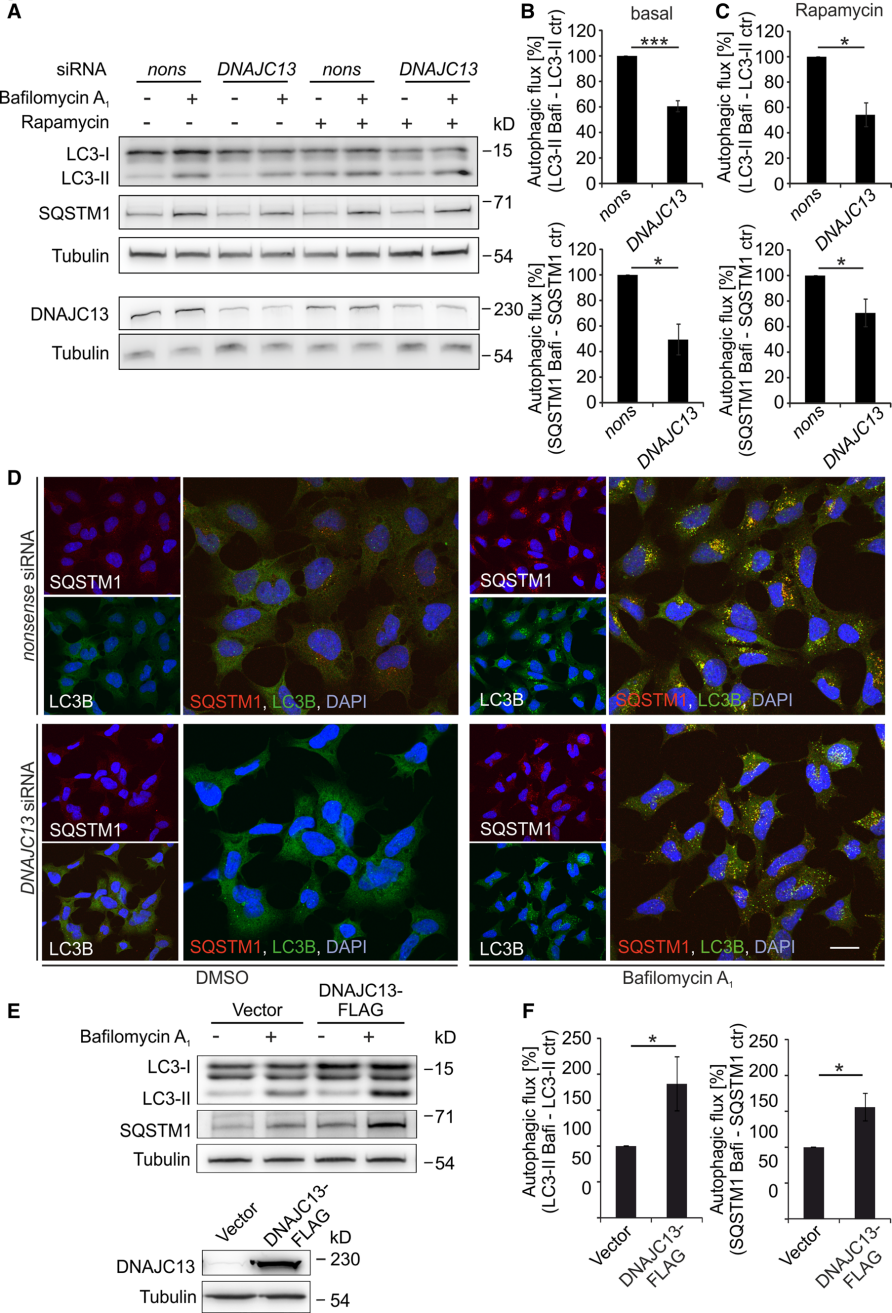
DNAJC13 positively modulates autophagy. **a**–**d** HEK293A cells were transfected with *nonsense* (*nons*) or *DNAJC13* siRNA. 48 h after transfection cells were treated with bafilomycin A_1_ and/or rapamycin for additional 4 h. **a**–**c** Cell extracts were separated on SDS-PAGE and transferred on nitrocellulose membrane. Expression levels of LC3B-II and SQSTM1 were quantified and normalized to tubulin. The lower band of the LC3B-I duplet in some blots may represent a processing intermediate [71]. Autophagic flux under basal conditions (**b**) or rapamycin treatment (**c**) was determined by subtraction of LC3B-II and SQSTM1 levels without bafilomycin A_1_ (ctr) from LC3-II and SQSTM1 levels with bafilomycin A_1_ (Bafi), respectively (*n* = 4 [basal], *n* = 3 [rapamycin]; mean ± SEM; *t* test: *p ≤ 0.05, ***p ≤ 0.001). **d** Confocal images of HEK293A cells transfected with *nonsense* or *DNAJC13* siRNA as above and stained for SQSTM1 and LC3B. The nucleus is detected by DAPI (scale bar: 20 µm). **e**, **f** Autophagic flux in HEK293T cells transiently overexpressing DNAJC13-FLAG was determined as in **a**, **b** (*n* = 4; mean ± SEM; *t* test: *p ≤ 0.05)

It has been well established that protein aggregates can be efficiently removed by autophagy and that, in turn, age-related or mutation-induced impairment of autophagic activity cause accumulation of misfolded proteins [6]. In addition, recent evidence that the precise interaction of the WASH and retromer complexes is required for autophagy [59] prompted us to investigate whether DNAJC13 is involved in autophagy. To determine autophagic flux, cells were exposed to bafilomycin A_1_, a compound that prevents the acidification of lysosomes and thereby leads to the accumulation of autophagosomes and autophagy targets, such as SQSTM1, an autophagy cargo receptor, and the lipidated form of LC3B, LC3B-II. When HEK293 or human primary fibroblasts (IMR90) were treated with *DNAJC13* siRNA, the autophagic activity was decreased compared to cells treated with *nonsense* siRNA (Fig. 2a–d; Suppl. Figure 2). Reduced DNAJC13 levels also led to lower autophagic flux under rapamycin treatment (Fig. 2a, c). The transient knockdown of *DNAJC13* did not alter the expression of genes involved in autophagy like *LC3B* or *SQSTM1*, nor were the protein levels of LC3B-I and SQSTM1 affected under basal conditions (Suppl. Figure 5A, B).

These data were independently confirmed in HeLa cells transiently transfected with GFP-LC3B, showing a reduced number of LC3B-positive puncta per transfected cell under basal conditions and after rapamycin treatment upon *DNAJC13* knockdown compared to controls (Suppl. Figure 3A–C). Furthermore, we employed RPE-1 cells stably expressing GFP-LC3B-RFP-LC3B(ΔG) [60] and determined the GFP/RFP ratio in individual cells upon *nonsense* and *DNAJC13* siRNA treatment and basal or starvation induced autophagy conditions. We observed that the GFP/RFP ratio was increased upon *DNAJC13* knockdown in either condition (Suppl. Figure 4) comparable to *ATG3* siRNA-treated cells, indicating that autophagy is inhibited.

### *DNAJC13* overexpression augments autophagic flux

Since knockdown of *DNAJC13* resulted in decreased autophagy, we investigated whether the overexpression of DNAJC13 might positively affect autophagic activity. Elevated protein levels of DNAJC13 resulted in an enhanced flux of LC3B-II and SQSTM1 compared to controls (Fig. 2e, f). The increased autophagic activity upon DNAJC13 overexpression was not mediated through an elevated transcription of autophagy-related genes like *ATG7*, *ATG3*, *LC3B,* or *SQSTM1* (Suppl. Figure 5A), despite LC3B-I and SQSTM1 protein levels were increased (Suppl. Figure 5C). To analyze if the direct interaction of DNAJC13 with phosphoinositides (PI), most prominently to PI(3)P, is relevant for this effect, we generated an FLAG-tagged mutant DNAJC13(W20A). This variant shows a dramatically reduced interaction with membranes [30]. The analysis of accumulated LC3B-II after bafilomycin A_1_ treatment suggested that DNAJC13(W20A) augmented autophagic flux comparable to increased levels of DNAJC13(WT) (Suppl. Figure 5D, E). In contrast, overexpression of the PD-related DNAJC13(N855S) mutant did not increase the autophagic activity (Suppl. Figure 5F, G), indicating an autophagy defect in Parkinson’s causing DNAJC13(N855S) mutant. It is of note that DNAJC13 itself is not degraded by autophagy as the protein levels of endogenous DNAJC13 as well as of DNAJC13-FLAG after overexpression were not significantly affected by bafilomycin A_1_ treatment (Suppl. Figure 5H, I).

### ATG9A subcellular distribution is altered upon DNAJC13 knockdown

As ATG9A is shuttling through the endosomal compartment [18, 20, 21, 23] and DNAJC13 is involved in the trafficking of endosomal cargos [31–34, 36], we analyzed whether DNAJC13 plays a role in ATG9A trafficking and investigated the subcellular distribution of ATG9A following *DNAJC13* knockdown. In *nonsense* siRNA-treated HEK293A cells, ATG9A was concentrated in a perinuclear compartment as described earlier [61] whereas the distribution was more diffuse in cells treated with siRNA against *DNAJC13* (Suppl. Figure 6A), pointing towards a change of the distribution of ATG9A-containing subcellular compartments. Total ATG9A protein levels were comparable between both conditions based on Western blot analysis (Suppl. Figure 6B). Moreover, we could demonstrate a partial co-localization of FLAG-tagged DNAJC13 with endogenous ATG9A by immunofluorescence analysis (Suppl. Figure 6C). It is of note that besides DNAJC13, also VPS35 partially co-localizes with ATG9A confirming the previous data [59]. The co-localization of DNAJC13 and ATG9A is further supported by experiments employing sucrose density gradient centrifugation. Endogenous DNAJC13 co-migrates in the same fractions as endogenous ATG9A as well as, in part, VPS35 (Suppl. Figure 6D). These data suggest that DNAJC13, ATG9A, and VPS35 reside within the same cellular compartment and that DNAJC13 interferes with ATG9A transport through the retromer and possibly the WASH complex [59].

**Fig. 3:**
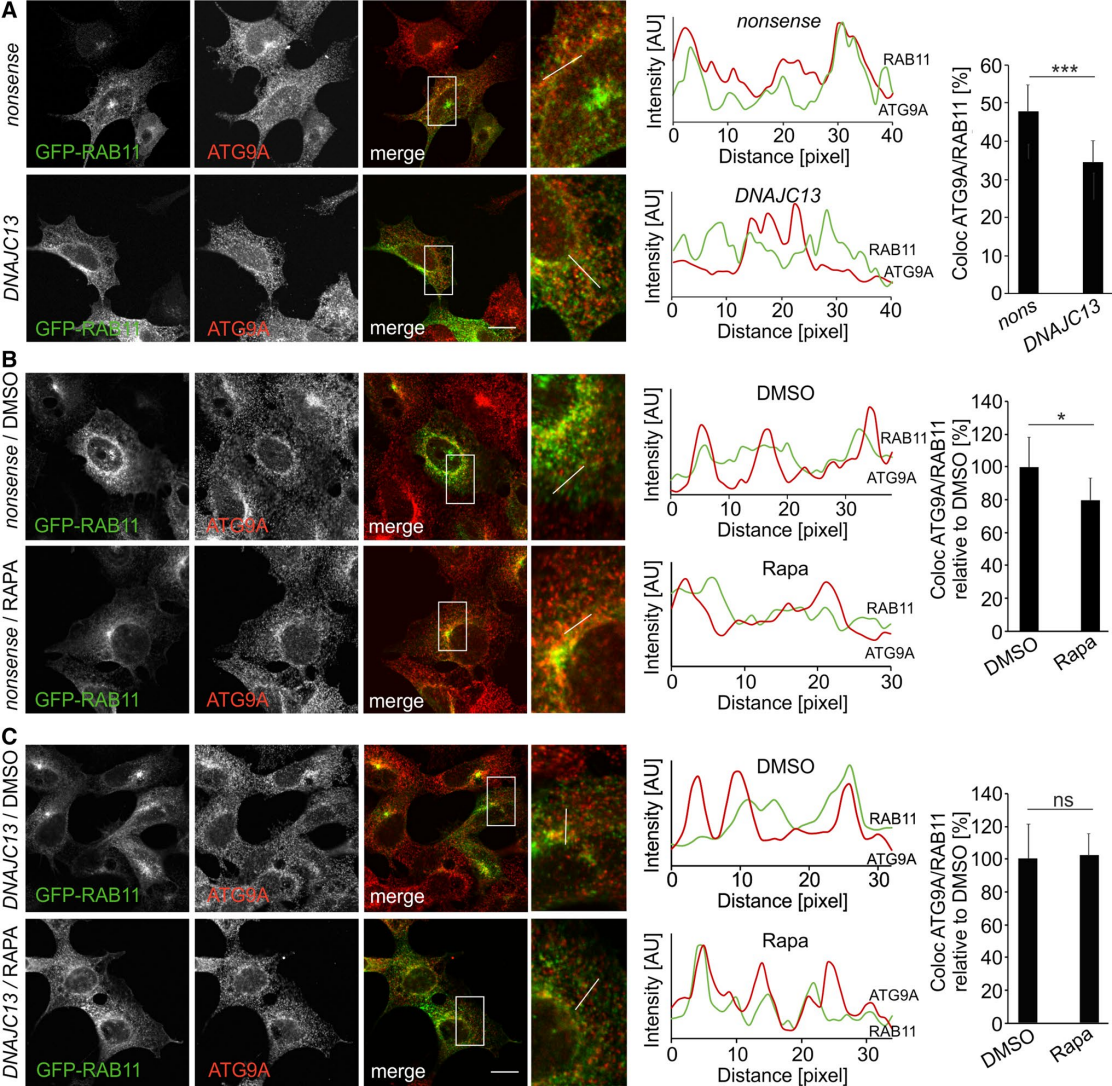
Knockdown of *DNAJC13* alters localization of ATG9A at the recycling endosome. Confocal images of HEK293A cells transfected with *nonsense* or *DNAJC13* siRNA and co-transfected with GFP-RAB11 under steady-state conditions (**a**) or rapamycin treatment (**b**, **c**). The box in each merged panel is enlarged and the fluorescent intensities along the white line are represented in the diagram, whereby ATG9A is shown in red throughout all panels. The overlap of ATG9A intensity peaks with GFP-RAB11 was quantified. The quantification method is described in detail in the Materials and Methods section. At least 13 regions of interest (ROIs) covering about 85 intensity peaks per cell were analyzed in 9–37 cells per condition (mean ± SD; *t* test: *p ≤ 0.05, **p ≤ 0.01, ***p ≤ 0.001) (scale bar: 15 µM)

To analyze ATG9A subcellular distribution in more detail, its localization in the trans-Golgi network, the late endosome, and the recycling endosome was studied by immunofluorescence labeling with anti-TGN46 antibodies or the expression of GFP-tagged RAB7 and RAB11, respectively. The co-localization of ATG9A to all three compartments was reduced upon *DNAJC13* knockdown under basal autophagy conditions (Fig. 3; Suppl. Figure 7A, B). The overall distribution of the trans-Golgi network (TGN46), the late (RAB7), and recycling (RAB11) endosome were not substantially altered when DNAJC13 levels were reduced. In contrast, the distribution of the mannose-6-phosphate receptor (CI-M6PR) which is transported in a DNAJC13-dependent manner is changed from a perinuclear staining to a more disperse localization throughout the cell (Suppl. Figure 8A), confirming earlier data [32]. Reduced DNAJC13 levels did not result in an untypical localization of ATG9A in lysosomes (LAMP2-positive compartment) (Suppl. Figure 8B) or the endoplasmic reticulum (CLIMP63-positive compartment) (Suppl. Figure 8C).

Upon autophagy induction, ATG9A vesicles shuttle between the trans-Golgi, the endosome, or the “ATG9 compartment” and the autophagosome precursor [61]. It was previously suggested that ATG9A does not integrate in the phagophore membrane, but ATG9A vesicles deliver membranes and important proteins towards the growing phagophore [18]. In this process, the routing of ATG9A through the recycling endosome is an essential step for proper autophagosome biogenesis [20–23]. After induction of autophagy with rapamycin in control cells (n*onsense* siRNA), ATG9A co-localization with RAB11-positive endosomal structures was reduced (Fig. 3a) which is consistent with earlier reports [21]. In contrast, co-localization under *DNAJC13* knockdown conditions was unchanged at low level after rapamycin treatment when normalized to DMSO-treated cells (Fig. 3b), indicating that DNAJC13 affects the trafficking of ATG9A to and from the recycling endosome.

Our data indicate that the modulation of autophagy by DNAJC13 is mediated through an altered ATG9A trafficking. This direct functional link between DNAJC13 and ATG9A was further supported by experiments where we assayed autophagic flux in cells overexpressing DNAJC13 upon transient knockdown of *ATG9A*. The reduction of ATG9A protein levels resulted in a comparable decrease of autophagic activity in control cells as well as in cells overexpressing DNAJC13 (Suppl. Figure 9B), suggesting that the effect of DNAJC13 overexpression depends on a sufficient amount of ATG9A.

### *DNAJC13* knockdown affects ATG16L1 trafficking and ATG9A co-localization with LC3B

**Fig. 4:**
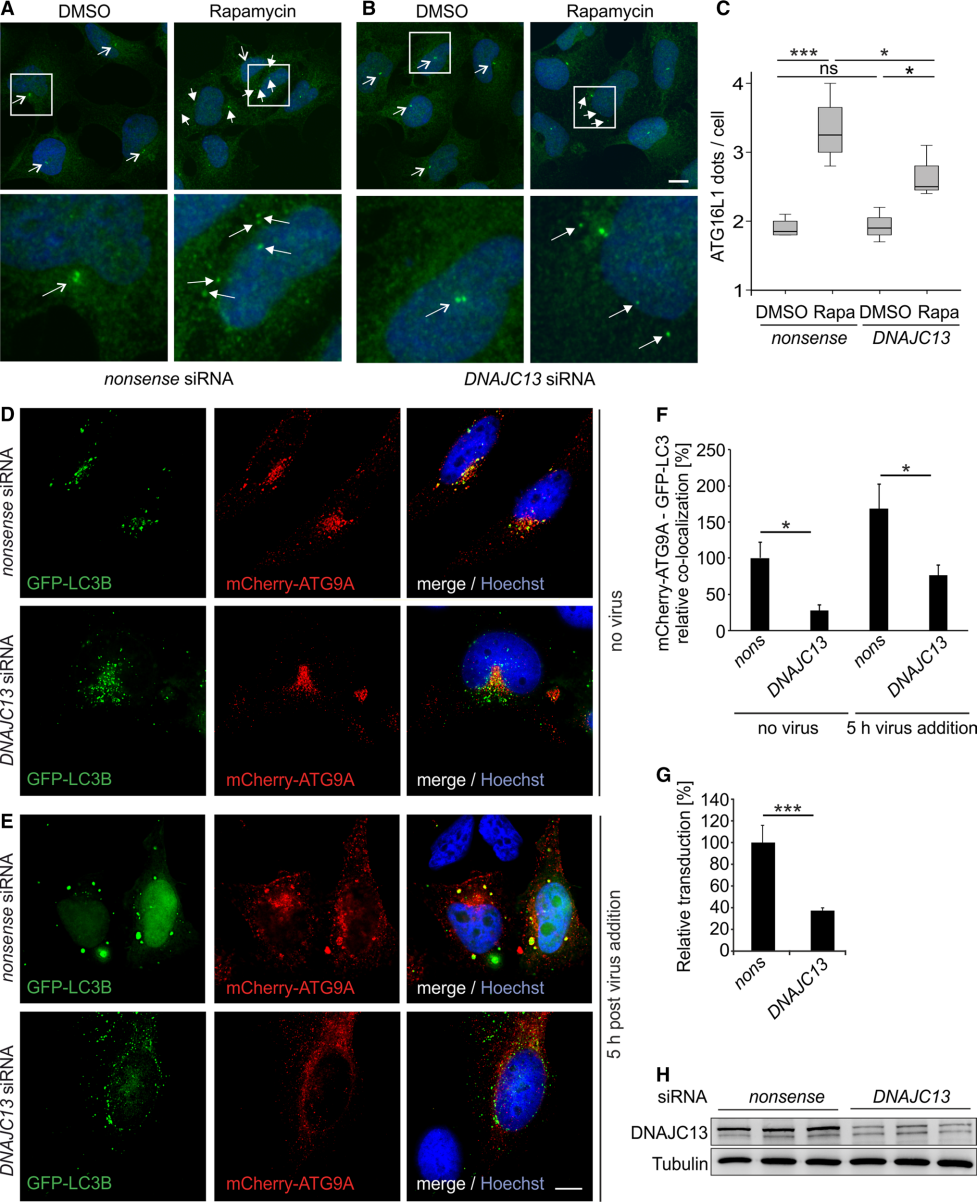
DNAJC13 affects the formation of ATG16L1 puncta and the co-localization of ATG9A with LC3. HEK293A cells transfected with **a** *nonsense* or **b** *DNAJC13* siRNA and treated with rapamycin were stained with antibodies detecting ATG16L1 (green) and DAPI (blue). Images represent maximum projections of image stacks. The lower panels are enlargements of the boxed areas. All cells carrying a single or a double point in close proximity to the nucleus (open arrows) represent cells with a low autophagic activity; multiple, accumulated ATG16L1 dots (closed arrows) reflect a high autophagic activity (scale bar: 10 µm). **c** In average, dots in 60 cells per condition and experiment were counted (*n* = 4; box-plot; one-way ANOVA with Bonferroni correction: *p ≤ 0.05; ***p ≤ 0.001). **d**, **e** HeLa cells were transfected with *nonsense* (*nons*) or siRNA against *DNAJC13* and GFP-LC3B (in green) and mCherry-ATG9A (in red). Representative deconvoluted images of z-stacks are shown without infection (**d**) and 5 h post-HPV pseudovirion addition (**e**). DNA was labeled with Hoechst and is shown in blue (scale bar: 10 µm). **f** Analysis of GFP-LC3B and mCherry-ATG9A resulted in a decreased co-localization of pixels upon knockdown of *DNAJC13*. The infection with HPV virions resulted in an increased co-localization of ATG9A and LC3B and the knockdown of *DNAJC13* affects this co-localization. About eight images per condition were analyzed in three independent experiments each (about 100 cells in total). Values were normalized to *nonsense* siRNA transfected cells without virus (*n* = 24; mean ± SEM; Wilcoxon rank-sum test: *p ≤ 0.05). **g** HeLa cells treated with *nonsense* or *DNAJC13* siRNA were infected with HPV virions containing a luciferase expression plasmid. Only cells in which the plasmid reached the nucleus expressed luciferase. Relative infection was measured by luciferase activity and normalized by LDH measurements. Control siRNA infection rate was set to 100% (*n* = 4; mean ± SD; *t* test: ***p ≤ 0.001). **h** DNAJC13 protein levels were determined after transfection of HeLa cells with *nonsense* or *DNAJC13* siRNA by Western blot. Tubulin served as a loading control

The recycling endosome serves as a hub for compiling ATG9A vesicles. One example for an uptake of cargo in ATG9A vesicles at the recycling endosome is ATG16L1, a protein required for LC3B lipidation. Although ATG9A and ATG16L1 enter the recycling endosome on different routes, the proteins traffic towards the phagophore in the same vesicles [62]. As ATG9A trafficking from the recycling endosome was impaired upon *DNAJC13* knockdown, we hypothesize that, as a consequence, the formation of autophagosomes is affected. To investigate this, we analyzed the trafficking of ATG16L1 and the co-localization of ATG9A with LC3B under autophagy-stimulating conditions. It had previously been shown that ATG16L1 is present at the phagophore, but not on autophagosomes [63]. To monitor ATG16L1 trafficking, we analyzed the number of ATG16L1-positive dots per cells under basal conditions and after autophagy stimulation with rapamycin. ATG16L1 was usually localized in one or two adjacent bright dots close to the nucleus (Fig. 4a, b). After autophagy induction, ATG16L1 dots were distributed in the cytosol. Most of these dots were WIPI2 positive (Suppl. Figure 9A), indicating that these represent phagophores. In this assay, less ATG16L1 dots per cell were counted under *DNAJC13* knockdown compared to control cells (Fig. 4a–c).

Comparable to ATG16L1, ATG9A is not integrated in the membrane of mature autophagosome, but a substantial portion is co-localized with LC3B upon autophagy induction [18]. We analyzed the localization of transiently expressed GFP-LC3B and mCherry-ATG9A upon *nonsense* or *DNAJC13* siRNA treatment under basal conditions and upon autophagy induction employing HeLa cells infected with human papillomavirus (HPV) [64]. Analysis of GFP and mCherry fluorescence revealed that LC3B and ATG9A were partially co-localized under control conditions which was significantly reduced (Fig. 4d, f) upon *DNAJC13* knockdown (Fig. 4h). When autophagy was stimulated by treatment with HPV pseudoviruses, a general increase in GFP-LC3B and mCherry-ATG9A co-localization was observed. In line with the data above, reduced DNAJC13 levels resulted in a significant decrease of LC3B and ATG9A co-localization compared to *nonsense* siRNA-treated cells after autophagy stimulation (Fig. 4e, f). It is of note that despite HPV virions were bound to and taken up by almost all HeLa cells independent of siRNA treatment, the delivery of virion DNA towards the nucleus and thereby the expression of luciferase as a marker protein was impaired in cells treated with *DNAJC13* siRNA (Fig. 4g).

## Discussion

We uncovered *rme-8*/*DNAJC13* as a novel factor to maintain proteostasis in *C. elegans* and in human cells. Upon reduction of RME-8/DNAJC13 protein levels, accumulation of aggregation-prone and age-related disease-causing proteins were found to be increased. As this phenotype points towards a defect in protein degradative pathways, we investigated the role of DNAJC13 in autophagy. Altogether, our data now provide first evidence that DNAJC13, but not the PD-related mutant variant DNAJC13(N855S), is a positive modulator of autophagy. Mechanistically, the lack of DNAJC13 interferes with the trafficking of ATG9A to and from the recycling endosome. As a consequence, the localization of ATG9A at LC3-positive-structures under steady-state and autophagy-induced conditions is impaired suggesting a functional cross-talk of DNAJC13 with this key autophagy protein.

RME8/DNAJC13 is primarily localized at the early endosome membrane which serves as a switchboard for protein and membrane traffic. RME-8/DNAJC13 affects retrograde, clathrin-mediated transport from endosomes towards the trans-Golgi network [33], endosomal tubulation [32], and the recycling of membrane receptors via the recycling endosome or through a direct route [36]. RME-8/DNAJC13 is linked to the retromer and the WASH complexes which represent molecular machineries for the sorting of endosomal proteins and the formation of distinct endosomal subdomains by forming actin-polymerization patches, respectively [65, 66]. The retromer complex consists of the classical cargo selection complex (VPS26, VPS35, and VPS29) that is linked to a sorting nexin dimer (SNX1/SNX2; SNX5/SNX6) [67]. The WASH complex is a protein pentamer that is involved in the formation of branched actin networks on endosomes [66]. By interacting with SNX1 [33, 34], a constituent of the sorting nexin dimer, and FAM21 [32], a component of the WASH complex, DNAJC13 is believed to orchestrate the interaction and thus the function of both complexes.

A coordinated action of WASH and retromer complexes has also been implicated in autophagosome formation by regulating ATG9A trafficking from endosomal membranes. ATG9A is an important component for the maturation of autophagosomes and the only transmembrane protein of the autophagy core machinery [17]. Upon autophagy induction, ATG9A vesicles shuttle between the trans-Golgi, the endosome, or the “ATG9 compartment” and the autophagosome precursor [61]. It was previously suggested that ATG9A does not integrate in the phagophore membrane, but ATG9A vesicles deliver membranes and other cargos towards the growing phagophore [18]. In this process, the routing of ATG9A through the recycling endosome is an essential step for proper autophagosome biogenesis [19–24].

**Fig. 5:**
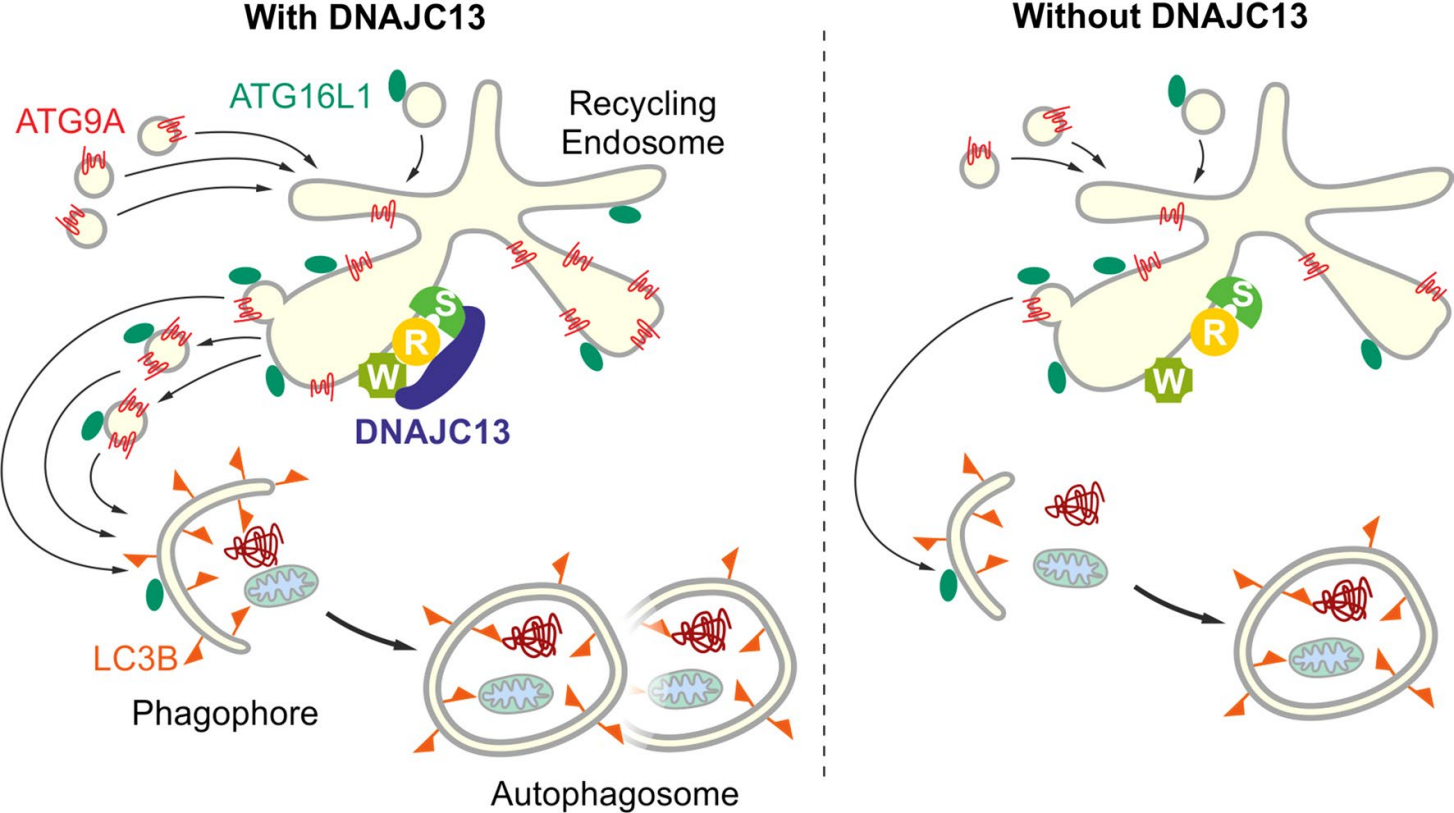
Proposed mode of action of DNAJC13 in autophagy by interfering with ATG9A trafficking. See details in the “Results” and “Discussion” section (*W* Wash complex, *R* retromer complex, *S* sorting nexin dimer)

The impairment of ATG9A localization and trafficking upon knockdown of *DNAJC13* is reminiscent of the ATG9A phenotype after the expression of the PD-related VPS35(D6120N) mutant. In these experiments, VPS35(D620N) did destabilize neither the retromer nor the WASH complexes [59, 68], but resulted in an impaired recruitment of the completely assembled WASH complex to endosomes which in turn altered ATG9A trafficking [59]. We now showed that the knockdown of DNAJC13 caused a similar phenotype than an impairment of endosomal WASH recruiting: reduced DNAJC13 levels (1) affected localization of ATG9A at the late and recycling endosomes, (2) impaired ATG9A transport from the recycling endosome upon autophagy stimulation, and (3) resulted in a reduced localization of ATG9A with LC3B under steady state and autophagy-stimulating conditions. It is of note that we additionally observed a reduced number of ATG16L1-positive puncta per cells after pharmacological autophagy induction in *DNAJC13* knockdown cells (summarized in Fig. 5). These data are consistent with the view that ATG16L1 gets hooked on ATG9A vesicles trafficking from the recycling endosome towards the site of autophagosome formation [62] and that an impaired ATG9A trafficking from the recycling endosome alters ATG16L1 localization at phagophores.

Several PD-causing mutations are located within genes like VPS35 [69, 70] and DNAJC13 [39] that facilitate endosomal protein trafficking. Increasing evidence suggests that mutant DNAJC13 expression results in an altered endosomal protein transport which affects also protein degradation. As an example, the expression of the DNAJC13(N855S) variant causes defects in SNX1 membrane dynamics [40] and actin cytoskeleton organization which is reminiscent of an disturbed WASH function [41]. In the latter, altered endosomal trafficking resulted in the accumulation of α-synuclein. In line with these experiments, we do not observe a significant activation of autophagic activity upon DNAJC13(N855S) expression compared to DNAJC13(WT) overexpression. This and the above-mentioned data indicate a dominant-negative mode of action of mutant DNAJC13(N855S) causing late-onset Parkinson’s disease. These data are consistent with the view that the chronic expression of factors challenging the autophagic pathway, like the expression of mutant variants of DNAJC13, results in increased proteotoxic stress that in turn significantly contributes to the development of late-onset neurodegenerative diseases.

## Electronic supplementary material

Below is the link to the electronic supplementary material.

Supplementary file1 (DOCX 10664 kb)

## Abbreviations

ATG: Autophagy-related
*C. elegans*: *Caenorhabditis elegans*
DNAJC: DnaJ-binding domain C
GFP: Green fluorescent protein
HEK: Human embryonic kidney
HPV: Human papilloma virus
HSC70: Heat shock cognate 70
LC3: Microtubule-associated protein 1 light chain 3
Luc: Luciferase
PI(3)P: Phosphatidylinositol-3-phosphate
RME-8: Receptor-mediated endocytosis 8
SNX18: Sorting nexin 18
SOD1: Cu/Zn-superoxide dismutase
SQSTM1: Sequestosome 1
TBC: Tre2/Bub2/Cdc16 domain
VPS: Vacuolar protein sorting
WASH: Wiskott–Aldrich syndrome protein and SCAR homolog
