# Supplementary material for: Receptor-mediated endocytosis 8 (RME-8)/DNAJC13 is a novel positive modulator of autophagy and stabilizes cellular protein homeostasis
Source: Cell Mol Life Sci. 2020 Apr 22;78(2):645–60. doi: 10.1007/s00018-020-03521-y (PMC7873018; doi:10.1007/s00018-020-03521-y)
Supplement: Supplementary file 1 — Supplementary file1 (DOCX 10664 kb) [file 18_2020_3521_MOESM1_ESM.docx]

**Receptor-mediated endocytosis 8 (RME-8)/DNAJC13 is a novel positive modulator of autophagy and stabilizes cellular protein homeostasis**

Anna S Besemer^1^, Joanna Maus^1^, Mirjam DA Ax^1^, Anna Stein^1^, Stella Vo^1^, Christian Freese^1^, Karsten Nalbach^1^, Christian von Hilchen^1^, Ines F Pfalzgraf^1^, Ingrid Koziollek-Drechsler^1^, Beate Silva^1^, Heike Huesmann^1^, Fatima Boukhallouk^2^, Luise Florin^3^, Andreas Kern^1^, Christian Behl^1,#^, Albrecht M Clement^1,#^

^1^Institute of Pathobiochemistry, ^2^Institute of Medical Microbiology and Hygiene, and ^3^Institute for Virology and Research Center for Immunotherapy (FZI), University Medical Center of the Johannes Gutenberg-University, Mainz, Germany

**Supplemental Material**

**Supplemental Methods**

*Cell culture*

Primary human fibroblast IMR90 cells were obtained from the Coriell Institute for Medical Research. Cells were maintained in high glucose-containing DMEM (Life Technologies, 41965062) supplemented with 10% fetal calf serum (PAA Laboratories, A15-101), 1 mM sodium pyruvate Life Technologies, 1136-088) , 1x non-essential amino acids (Life Technologies, 15140122) and 1x antibiotics-antimycotics (Life Technologies, 155240-112) at 37°C and 5% CO_2_ in a humidified atmosphere.

For transient transfection using the calcium-phosphate precipitation method, cells were seeded on plastic dishes 24 h prior to transfection with fresh medium. For each transfection, 10 μg of plasmid were mixed with 420 μl ddH_2_O and 60 μl 2 M CaCl_2_. To this solution 480 μl transfection buffer (280 mM NaCl, 50 mM HEPES, 1.5 mM Na_2_HPO_4_; pH 7.12) were added dropwise while gently agitating. This mixture was incubated 30 minutes at room temperature before adding it to a single cell culture dish. 24 h after transfection, cells were washed with DMEM to remove precipitates and fresh medium was added.

*Virus production for stable Luc-GFP expressing HEK293A cells*

Virus-producing GP2 cells were transfected with pLHCX:Luc-GFP by Fugene (Promega, E2312) according to the manufacturer’s protocol. 24 h after transfection, the medium was changed and medium was collected after an additional 24 h incubation. Medium was cleared by centrifugation and the virus-containing supernatant was incubated with HEK293A cells for 36 h, followed by an incubation of transfected cells in complete medium containing 250 µg/ml hygromycine (Life Technologies, 10687010) to select for stably transfected cells.

*Cell fractionation assay*

Cells expressing α-synuclein or SOD1(G85R) were extracted in PBS supplemented with protease inhibitors and sonicated. Lysates were separated by centrifugation (15,500 x*g*, 4°C, 15 min) in a soluble and a pellet fraction. The pellet was washed and resuspended in SDS-containing lysis buffer. Equal amounts of proteins were separated by SDS-PAGE under denaturing conditions. Proteins were transferred on nitrocellulose membrane and proteins were detected by specific antibodies.

*Quantitative real-time PCR*

RNA from transfected cells was purified with the NucleoSpin II Kit (Macherey-Nagel, 740955; Agilent Technologies, 400800) according to the manufacturer’s protocol. cDNA was produced with the Omniscript RT Kit (Qiagen, 205113) and immediately processed for quantitative real-time PCR using an iCycler or the CFT Connect system (Bio-Rad). The primer mix (Suppl. Table 2) was supplemented with SensiFast SYBR green (Bioline, Bio-96020) for quantitative DNA measurement. The specificity of the reaction was determined by monitoring the melting curves. Samples were measured in triplicates and data were analyzed with the REST software^1^ or the CFX Maestro Analysis Software (Bio-Rad) based on the ΔΔCt method.

*Analysis of RPE-1 cells expressing GFP-LC3B-RFP-LC3B(ΔG)*

RPE-1 cells (kind gift from I. Dikic, Frankfurt, Germany) stably expressing GFP-LC3B-RFP-LC3B(ΔG) were cultivated in DMEM-F12 medium (Lonza, BE12-719F), supplemented with 10% FCS, 1% penicillin-streptomycin, together with 0.02mg/ml hygromicin + 1mg/ml neomycin + 2ng/ml puromycin at 37°C in 5% CO_2_ in an humidified atmosphere. Cells were transfected with *DNAJC13* and *ATG3* siRNAs by Lipofectamine RNAiMAX (ThermoFisher, 13778030). 48 h past transfection, cells were starved with EBSS (Sigma, E2888) or cultured in full medium for 6 h. Cells were either harvested for mRNA purification, Western blot analysis, or fixed with 4% paraformaldehyde for immunofluorescent analysis. Confocal images for a particular experiment were taken with the same settings for all conditions whereby the signals were not overexposed. Individual cells were outlined and intensities were quantified with the FIJI software. About 250 cells per condition from four individual experiments were analyzed.

*Sucrose density gradient centrifugation*

HeLa cells were processed for sucrose density gradient centrifugation as described elsewhere (Scheffer et al., 2013). In brief, HeLa cells (confluent 150 cm^2^ dish) were extracted by passing though a 22-G needle. The post-nuclear supernatant (PNS) was adjusted to 40.6% sucrose and about 1 ml was loaded at the bottom of a centrifuge tube. The PNS was overlayed with 1.5 ml of a 35%, 1 ml of a 25% sucrose solution and homogenization buffer to fill up the tube. After centrifugation (14,000 x*g* for 1 h at RT) in a SW60 rotor (Beckman), 300 µl fractions were harvested from the top of the gradient. 40 µl per fraction were separated by SDS-PAGE followed by Western blot. It is of note that the samples were heated to 60°C for 5 min before loading on the gels.

**References**

1. Pfaffl MW, Horgan GW, Dempfle L. Relative expression software tool (REST) for group-wise comparison and statistical analysis of relative expression results in real-time PCR. Nucleic Acids Res 2002;30:e36.

2. Scheffer KD, Popa-Wagner R, Florin L. Isolation and characterization of pathogen-bearing endosomes enable analysis of endosomal escape and identification of new cellular cofactors of infection. Methods Mol Biol. 2013;1064:101-13. doi: 10.1007/978-1-62703-601-6_7.

**Supplemental Table 1: siRNA**

| **Target** | **Sequence (5‘ – 3‘)** |
| --- | --- |
| *DNAJC13* #1 | UUAUGACUACACACUAGAA |
| *DNAJC13* #2 | UGCCUAGCAUCAUCAAGGA |
| *ATG3* #1 | GAUGGAAUAUUCAGAUGAA |
| *ATG3* #2 | UGUAAUGAAGAGAGCAUAA |
| *HSC70* | GAACUGAAUAAGAGCAUCA |
| *HSF1* | CAGGUUGUUCAUAGUCAGAAU |
| *GAPDH* | CGGGAAGCUCACUGGCAUG |
| *nonsense* | GGGAAAUGCGUUAGCAGUGAU |

**Supplemental Table 2: Oligonucleotides for RT-PCR**

| **Target** | **Sequence (5‘ – 3‘)** |
| --- | --- |
| *C. elegans* |  |
| RT-PCR-rme-8.for | GCTGAAGCTGGTTTGGAAAG |
| RT-PCR-rme-8.rev | CATTTGGCCGTACTCGATTT |
| RT-PCR-rpl-21.for | CCAGTCCCAGCTTTGAAGAG |
| RT-PCR-rpl-21.rev | ACAATCTCGAAACGGAGTGG |
|  |  |
| Human cell lines |  |
| RT-PCR-Atg3.for | GATGGCGGATGGGTAGATACA |
| RT-PCR-Atg3.rev | TCTTCACATAGTGCTGAGCAATC |
| RT-PCR-Atg4b.for | ATGGACGCAGCTACTCTGAC |
| RT-PCR-Atg4b.rev | TTTTCTACCCAGTATCCAAACGG |
| RT-PCR-Atg7.for | TGGAACAAGCAGCAAATGAG |
| RT-PCR-Atg7.rev | AGACAGAGGGCAGGATAGCA |
| RT-PCR-LC3B.for | CTGTTGGTGAACGGACACAG |
| RT-PCR-LC3B.rev | CTGGGAGGCATAGACCATGT |
| RT-PCR-p62.for | GACTACGACTTGTGTAGCGTC |
| RT-PCR-p62.rev | AGTGTCCGTGTTTCACCTTCC |
| RT-PCR-HSC70.for | TTCTTTGCGGCATCACCGATCAAC |
| RT-PCR-HSC70.rev | CTGGCCATGAAGCATGAGAA |
| RT-PCR-HSF1.for | CTGGCCATGAAGCATGAGAA |
| RT-PCR-HSF1.rev | GAGAACTGCCGGCTATACTT |
| RT-PCR-GAPDH.for | GCATCTTCTTTTGCGTCG |
| RT-PCR-GAPDH.rev | TGTAAACCATGTAGTTGAGGT |
| RT-PCR-RPL19.for | GAAATCGCCAATGCCAACTC |
| RT-PCR-RPL19.rev | TTCCTTGGTCTTAGACCTGCG |

**Supplemental Figures**

**
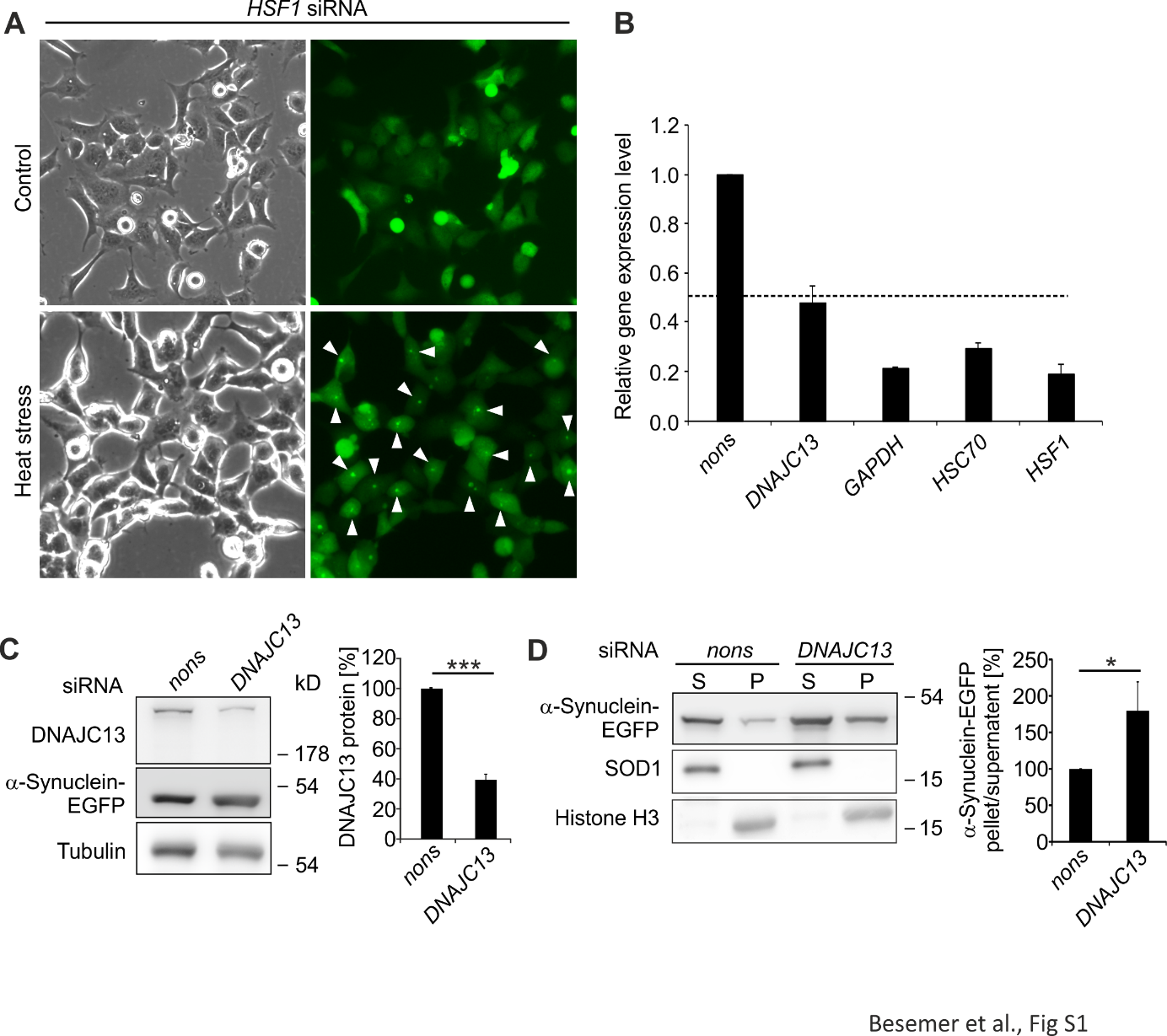
**

**Supplemental Figure 1: Knockdown of *DNAJC13* modulates aggregation of α-synuclein .** (A) Phase-contrast and epi-fluorescent images of HEK293A stably expressing Luc-GFP under control conditions or after heat stress and *HSF1* siRNA treatment. Quantification of the punctae is shown in Fig. 1. (B) The efficiency of the siRNAs was monitored by qPCR and normalized to *nonsense* siRNA treated cells. Relative expression levels below 0.5 are considered to be significant (n=3; mean+/-SEM). (C, D) HEK293T cells were transfected with a construct expressing EGFP-tagged *a*-synuclein and *nonsense* siRNA (*nons*) or *DNAJC13* siRNA. (C) The protein levels of DNAJC13 and α-synuclein in the total lysate were analyzed by Western blot. DNAJC13 levels were normalized to tubulin. (D) Lysate were separated in a soluble and a pellet fraction by centrifugation. Equal amounts of protein were analyzed by Western blot and the ratio between supernatant and pellet was calculated. SOD1 and histone H3 served as controls for the soluble and the aggregate-enriched fractions, respectively (n=4; mean+/-SD; t-test: *p≤0.05; ***p≤0.001)


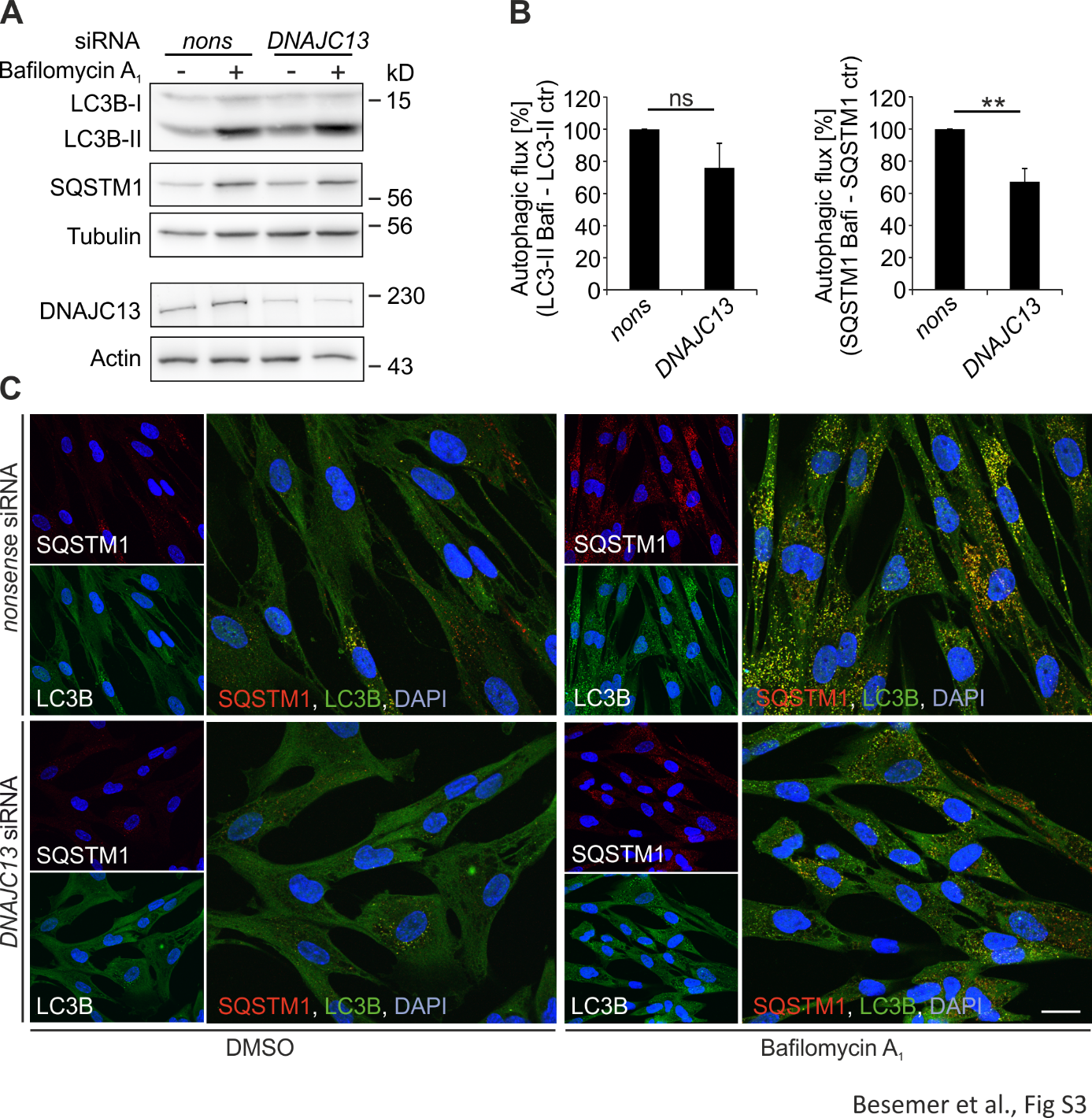


**Supplemental Figure 2: Knockdown of *DNAJC13* affects autophagy in primary fibroblasts.** IMR90 primary fibroblasts were transfected with *nonsense* siRNA or *DNAJC13* siRNA and treated with bafilomycin A_1_ to analyze the autophagic flux. (A,B) Cell extracts were analyzed by Western blot and protein levels of LC3B-II and SQSTM1 were quantified and normalized to tubulin. The autophagic flux was determined by subtraction of LC3B-II and SQSTM1 levels of control cells (ctr) from LC3B-II and SQSTM1 levels of bafilomycin A_1_ (Bafi) treated cells, respectively (n=5; mean+/-SEM; t-test: **p≤0.01). (C) Confocal images of IMR90 cells treated with *nonsense* or siRNA to *DNAJC13* and exposed to bafilomycin A_1_. Autophagosomes were detected by antibodies against LC3B and SQSTM1 with corresponding fluorescent secondary antibodies. The nucleus was stained with DAPI (scale bar: 20 µm).


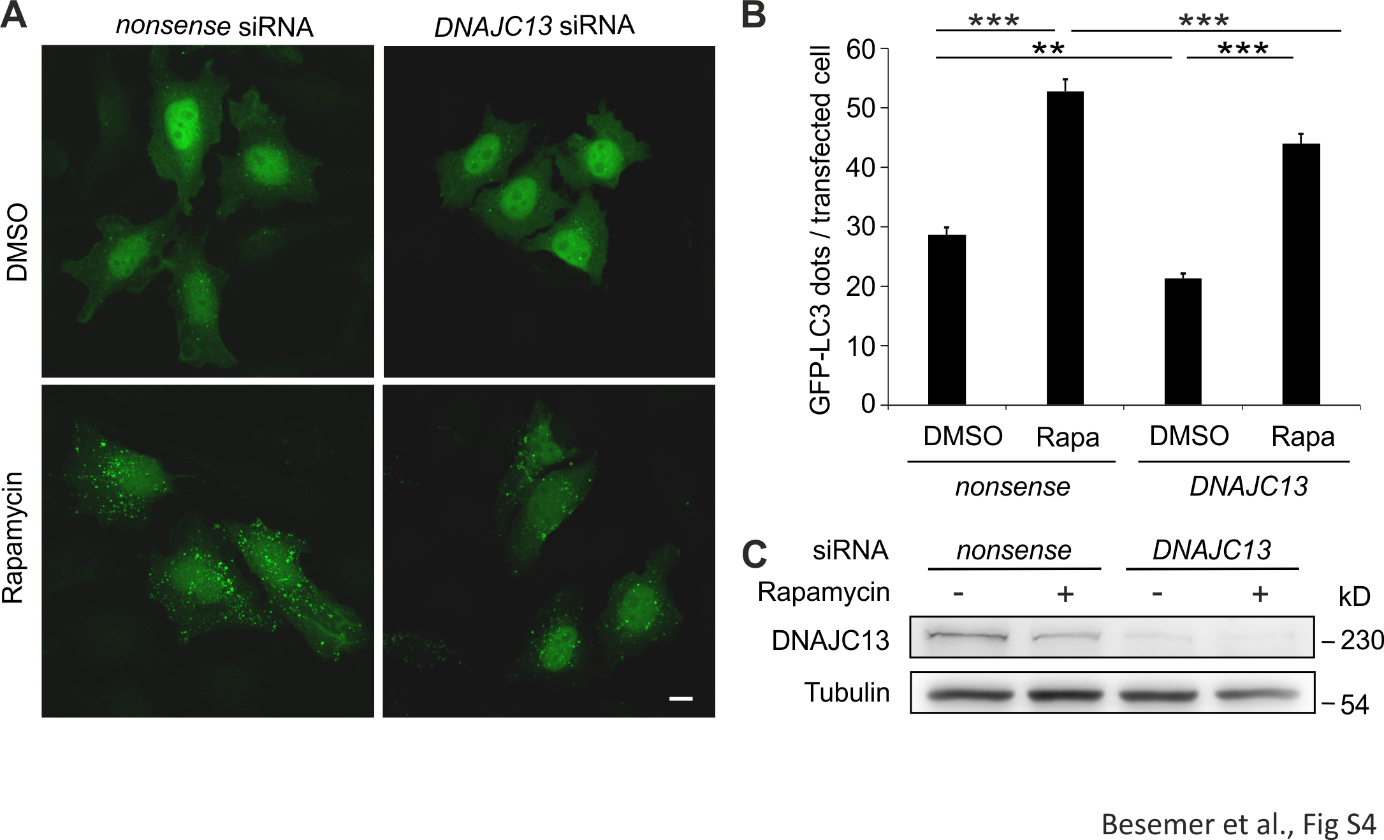


**Supplemental Figure 3: DNAJC13 affects the formation of LC3B-positive puncta.** HeLa cells were transfected with GFP-LC3B and *nonsense* siRNA or *DNAJC13* siRNA and treated with rapamycin. Maximal intensity projections of confocal stacks show the appearance of GFP-LC3B-positive puncta under control conditions and rapamycin treatment (scale bar: 20 µm). (B) The number of GFP-LC3B dots per cell was determined in transfected cells. About 150 cells out of three independent experiments were analyzed. (n=3; mean+/-SEM; one-way ANOVA with Games-Howell correction: **p≤0.01; ***p≤0.001). (C) The level of DNAJC13 in transfected cells were determined by Western blotting within each experiment. One representative Western blot is shown.


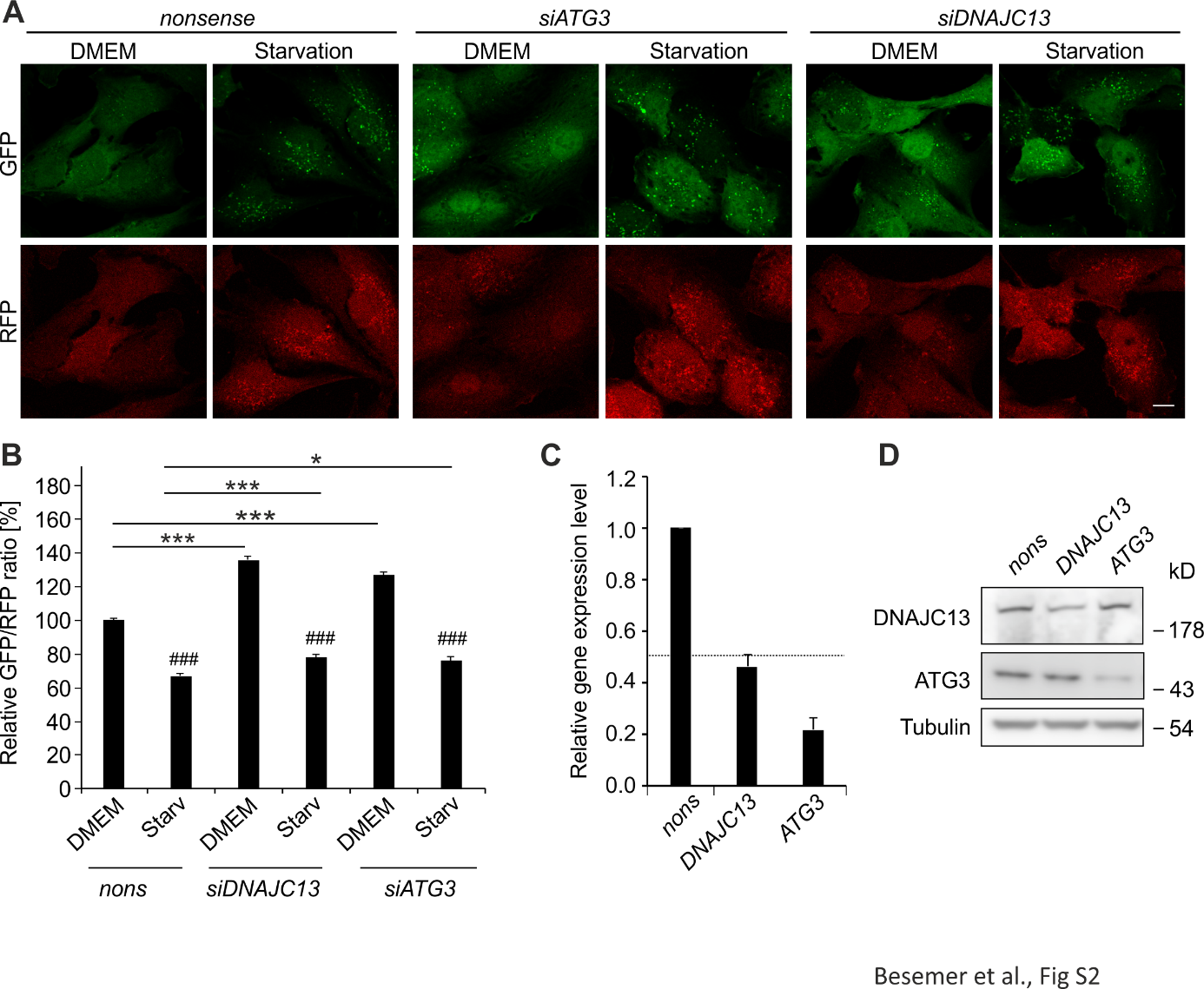


**Supplemental Figure 4: Knockdown of *DNAJC13* and *ATG3* impairs autophagic flux.** RPE-1 cells stably expressing GFP-LC3B-RFP-LC3B(ΔG) were treated with *nonsense*, *DNAJC13*, or *ATG3* siRNA. 48 h past transfection, cells were cultivated with full medium (DMEM) or starved (Starv) for 6 h. (A) Confocal images show GFP and RFP signals. Images of one experiment were taken with the same settings. (B) GFP and RFP fluorescent intensities of about 250 cells per condition out of four independent experiments were determined, the GFP/RFP ratios were calculated, and normalized to control conditions (n=4; mean+/-SEM; one-way ANOVA with Games-Howell corrections: *p≤0.05; **p≤0.01; ***p≤0.001; #: significance to DMEM conditions). (C) The efficiency of the knockdown was determined by qPCR (ΔΔCt values) and normalized to control cells (n=3; mean+/-SEM). (D) Representative Western blot of total RPE-1 cell lysates to monitor reduced DNAJC13 and ATG3 protein levels upon *DNAJC13* and *ATG3* siRNA treatment, respectively.


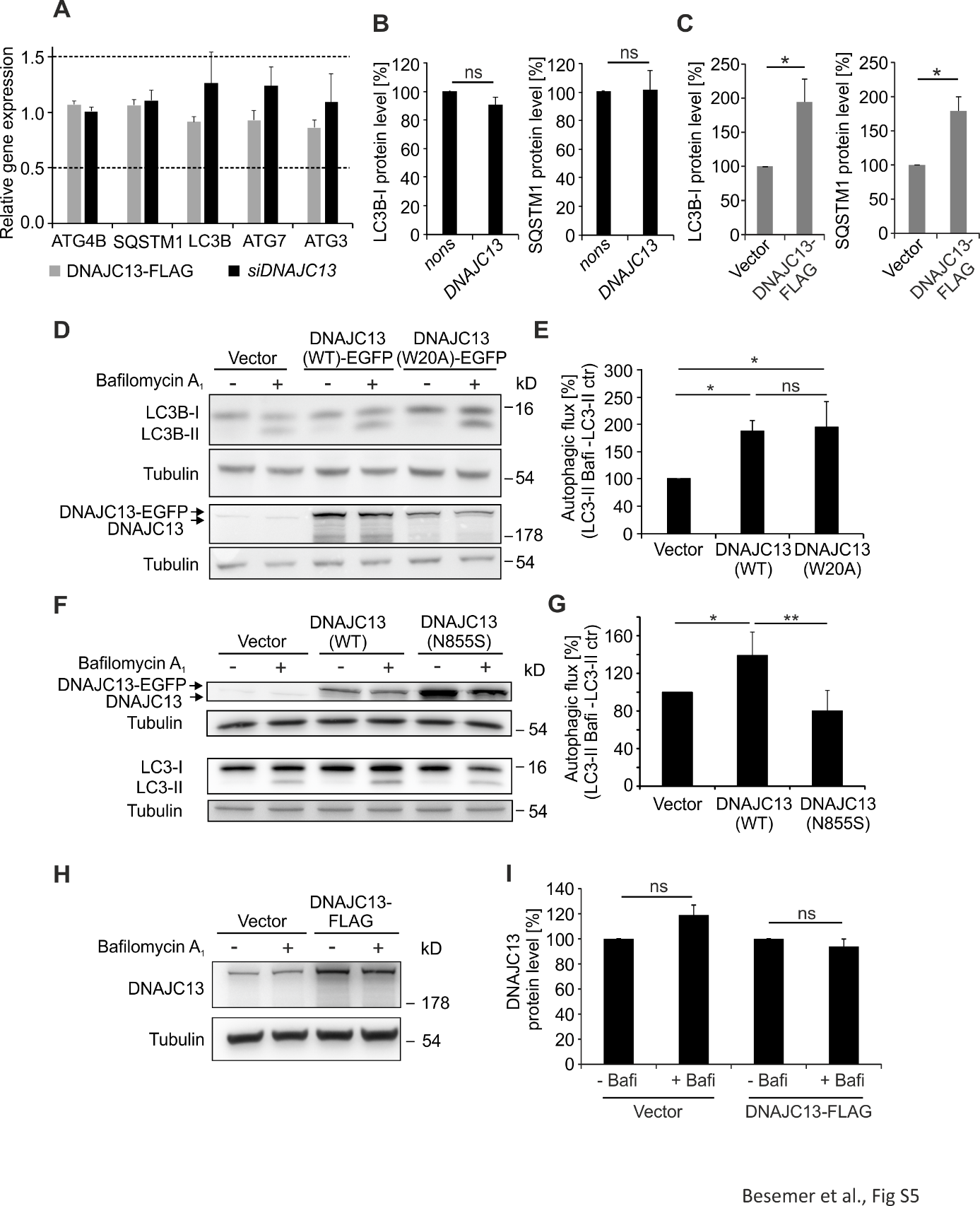


**Supplemental Figure 5: Overexpression of DNAJC13 and DNAJC13(W20A), but not DNAJC13(N855S), increases autophagic flux.** (A) HEK293T cells were transfected with the DNAJC13-FLAG construct (or empty vector) or *DNAJC13* siRNA (or *nonsens*e). mRNA was isolated and mRNA levels of *ATG4b*, *SQSTM1*, *LC3B*, *ATG7*, and *ATG3* were determined by quantitative qPCR detecting SYBR-green. Bars show values normalized to the corresponding control. Statistical analysis revealed no significant alteration of mRNA levels from treated cells compared to the corresponding controls (n=3; mean+/-SEM; t-test). (B,C) Protein levels of LC3B-I and SQSTM1 under basal conditions were quantified from experiments shown in Fig 2A,E. (B) The knockdown of *DNAJC13* did not change protein levels of LC3B-I and SQSTM1 under basal conditions. (C) The overexpression of DNAJC13 resulted in elevated LC3B-I and SQSTM1 protein levels. (D-G) HEK293T cells transiently overexpressing DNAJC13(W20A)-FLAG (D,E) and DNAJC13(N855S)-EGFP (F,G) were treated with bafilomycin A_1_. Protein levels of LC3B-II, SQSTM1 and DNAJC13 were determined by Western blotting. Autophagic flux was determined by subtracting LC3B-II and SQSTM1 levels under control conditions (ctr) from levels of bafilomycin A_1_ (Bafi) treated. [DNAJC13(W20A): mean+/-SD; n=3; one-way ANNOVA with Bonferroni correction; *p≤0.05; ns: not significant); DNAJC13(N855S):(n=5; mean+/-SEM; one-way ANOVA with Bonferroni post-hoc correction: *p≤0.05, **p≤0.01)]. (H,I) HEK293T cells were transfected with empty vector (Vector) or a plasmid carrying DNAJC13-FLAG cDNA and treated with bafilomycin A_1_. DNAJC13 protein levels were determined by Western blot (H) and analyzed by densitometry (I). Neither endogenous DNAJC13 nor overexpressed DNAJC13-FLAG are significantly increased after bafilomycin A_1_ treatment (n=4; mean+/-SEM; t-test: ns not significant).


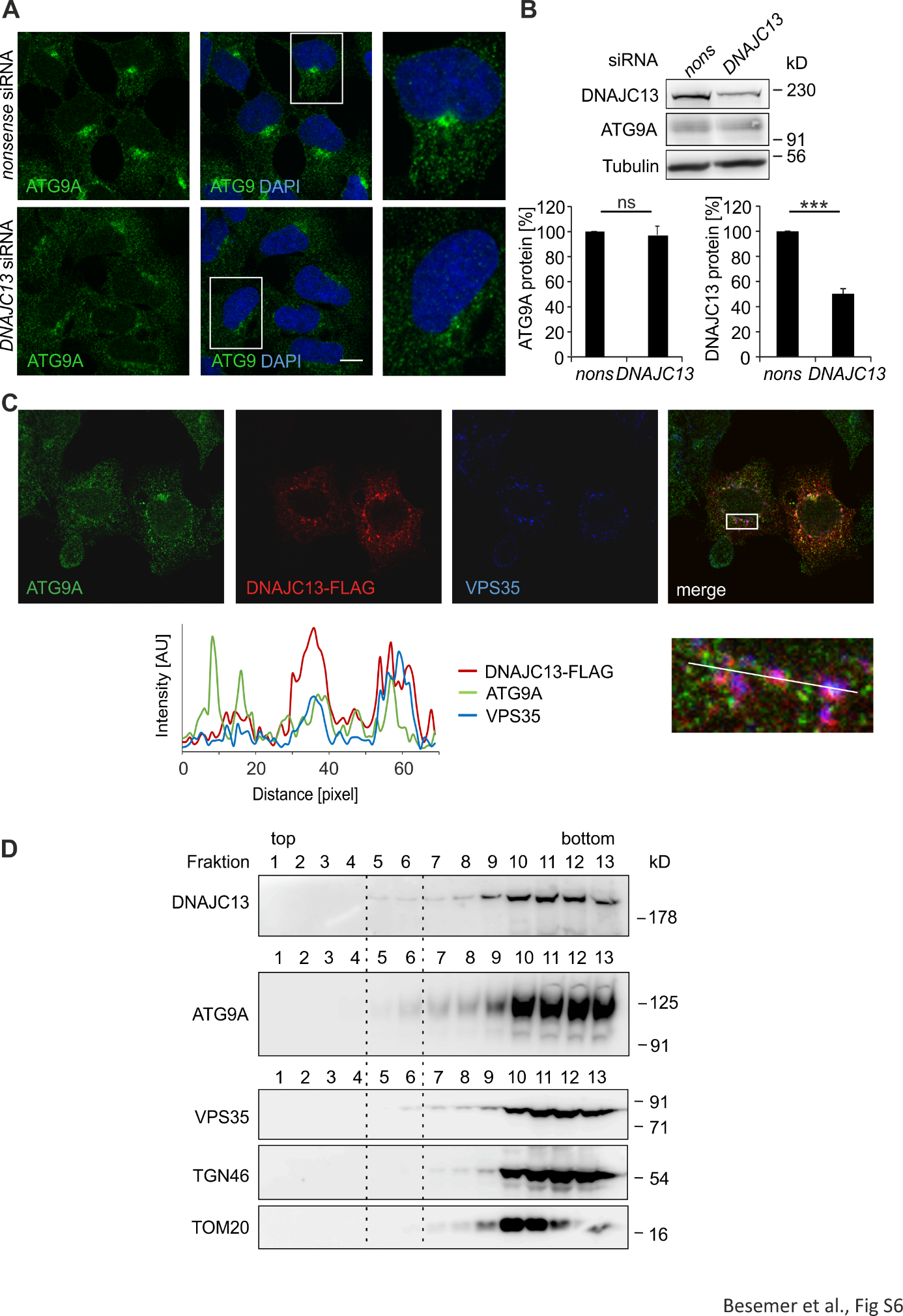


**Supplemental Figure 6: ATG9A localization is altered upon *DNAJC13* knockdown.** (*A*,B) HEK293A cells were transfected with *nonsense* (*nons*) or *DNAJC13* siRNA and the distribution (A) and protein levels of ATG9A (B) were analyzed. (A) Confocal images of ATG9A immunostainings of transfected HEK293A cells. Nuclei of cells were counterstained with DAPI (blue) (scale bar: 10 µm). (B) Protein levels of DNAJC13 and ATG9A were determined by Western blotting and normalized to tubulin (n=3; mean+/-SEM; t-test: ***p≤0.001). (C) HEK293A cells were transfected with a DNAJC13-FLAG plasmid. Antibodies against FLAG, VPS35, and ATG9A were visualized by species-specific fluorescent secondary antibodies. The box in the merge panel is enlarged and the fluorescent intensities along the line are represented in the diagram (DNAJC13-FLAG in red; ATG9A in green; VPS35 in blue). (D) HeLa cells were homogenized in a detergent-free buffer and the post-nuclear supernatant was applied to sucrose density gradient centrifugation whereby about 1 ml of sample was loaded at the bottom of the tube (representing fraction 10 to 13). Fractions of 300 µl were collected from the top and 40 µl were analyzed by Western blotting. DNAJC13 and ATG9A were separated on different 8% SDS-PAGE gels, VPS35, TGN46, and TOM20 were detected on the same blot (12% SDS-PAGE gel).


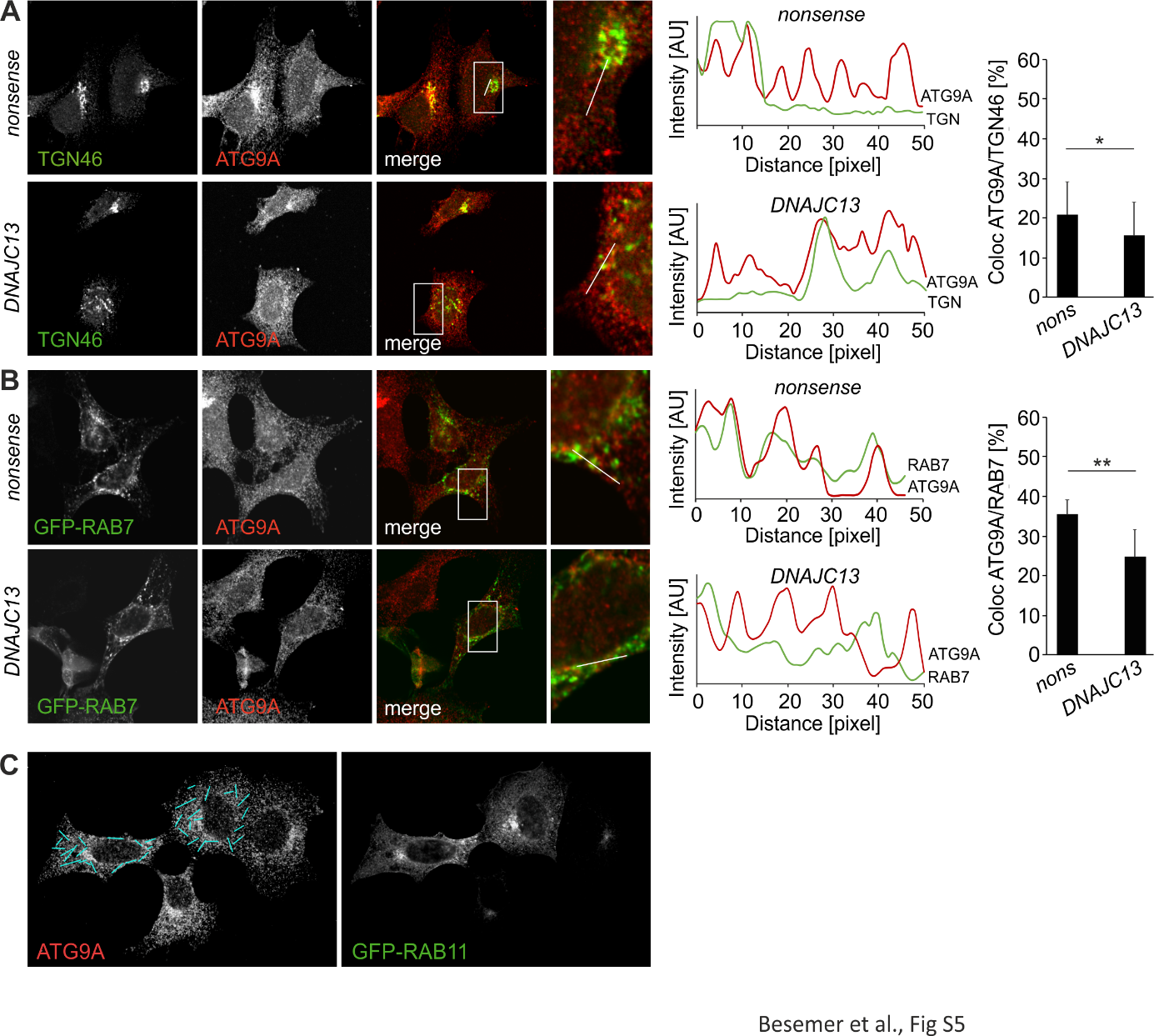


**Supplemental Figure 7: The localization of ATG9 at the late endosome and the trans-Golgi network is reduced with *DNAJC13* knockdown.** (A,B) Confocal images of HEK293A cells transfected with nonsense or DNAJC13 siRNA under steady-state conditions. Cells were stained for ATG9A and (A) trans-Golgi network protein 2 (TGN46) or (B) co-transfected with GFP-RAB7 to label the trans-Golgi network and the late endosome, respectively. The box in each merged panel is enlarged and the fluorescent intensities along the white line are represented in the diagram whereby ATG9A is shown in red throughout all panels. The overlap of ATG9A intensity peaks with the subcellular markers was quantified. The quantification method is described in detail in the Material and Method section. At least 13 regions of interest (ROIs) covering about 85 intensity peaks per cell were analyzed in 9 to 37 cells per condition (mean+/-SD; t-test: *p≤0.05, **p≤0.01, ***p≤0.001) (scale bar: 15 µM). (C) Representative images demonstrating the co-localization analysis. In this case, HEK293A cells were transiently transfected with GFP-RAB11 and stained with ATG9 and species-specific Cy3-conjugated secondary antibodies. Only cells with a dotted distribution of GFP-RAB11 were analyzed. Unprocessed GFP and Cy3 confocal single slice images were analyzed with the FIJI software using the plot profile tool. Over all, at least 13 lines resulting in about 85 punctae per cell and between 9 and 37 cells per condition were analyzed. Subsequently, intensity profiles were compared with GFP signals on the very same lines and overlapping peaks were counted.


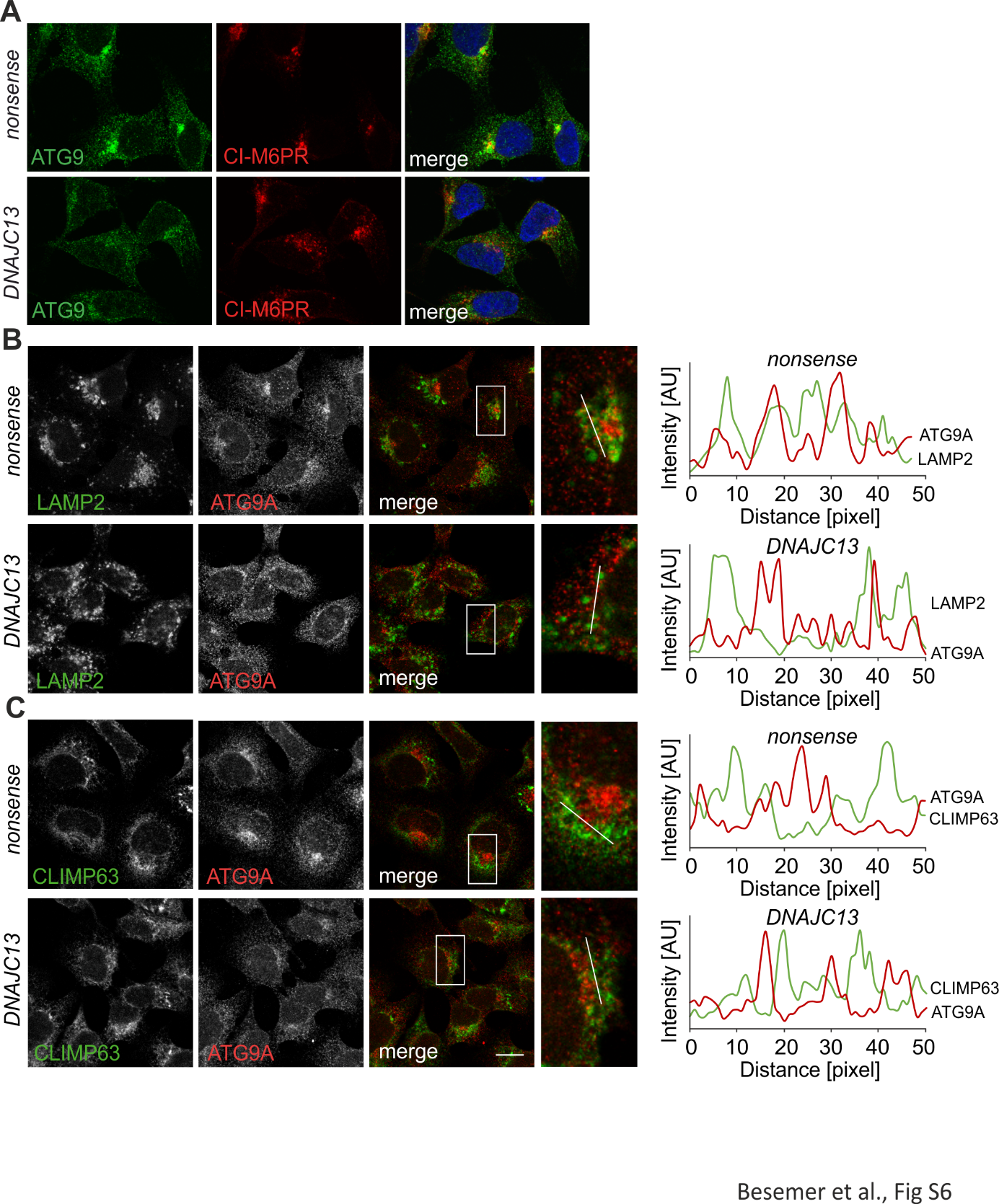


**Supplemental Figure 8: The localization of ATG9 with lysosomes and the ER is independent of DNAJC13.** (A) HEK293A cells were transfected with *nonsense* or *DNAJC13* siRNA. After 48 h cells were fixed and stained for ATG9 (green) and cation-independent mannose-6-phosphat receptor (CI-M6PR, red). (B,C) HEK293A cells were treated as in (A) and LAMP2 (B) (green; lysosomes), or CLIMP63 (C) (green, endoplasmic reticulum) were detected by specific antibodies. The boxes in the merge panel were enlarged and the fluorescent intensities along the white bar were determined.


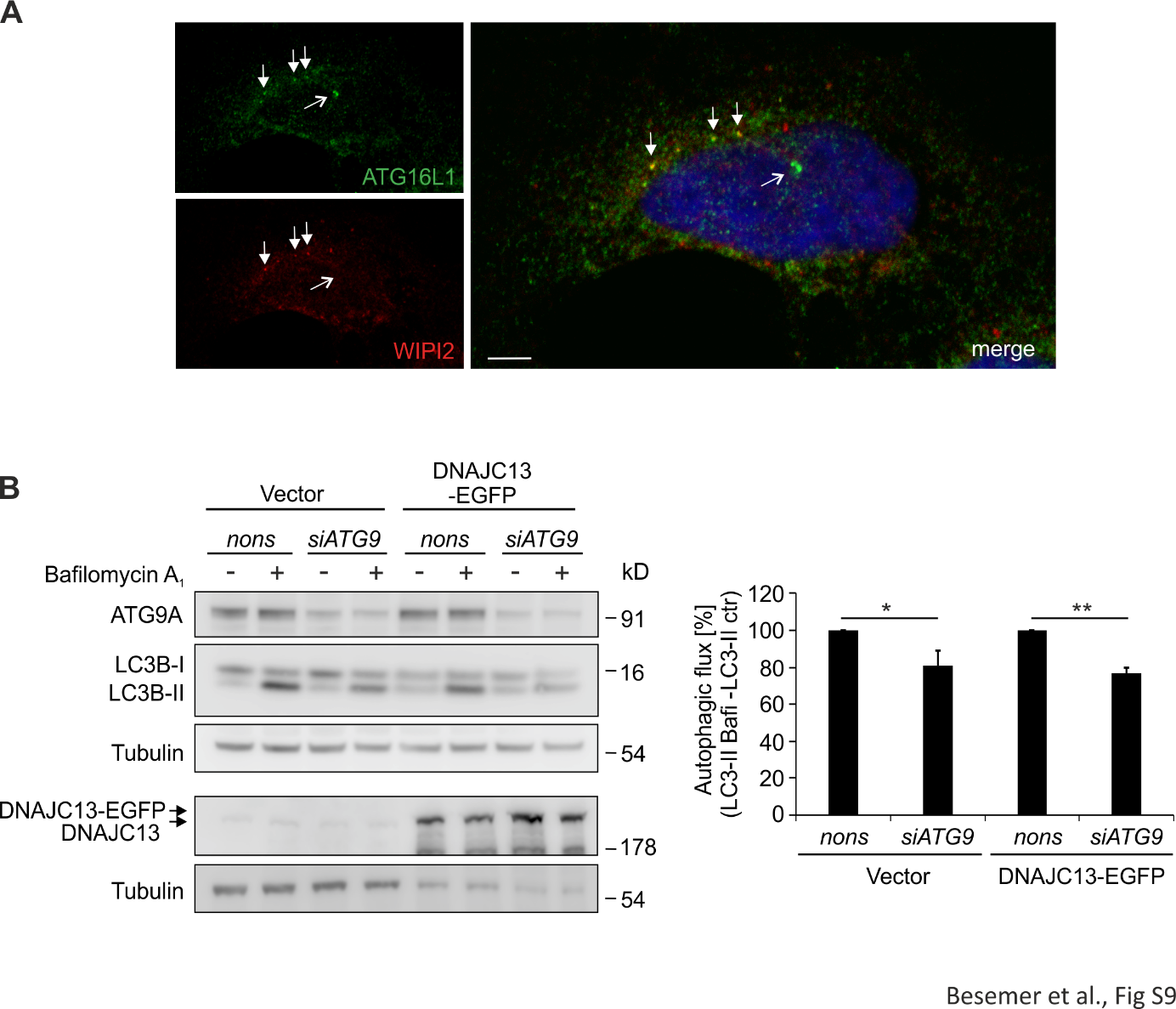


**Supplemental Figure 9: Knockdown of ATG9A impairs autophagic flux.** (A) HEK293A cells were transfected with *DNAJC13* siRNA and treated with rapamycin (see Fig. 4). Cells were stained with specific antibodies against ATG16L1 (green) and WIPI2 (red). Image represents a maximum intensity projection of a confocal image stack (scale bar: 5 µm). (B) Hek293T cells were transfected with DNAJC13-EGFP or a vector control and with *nonsense* or *ATG9A* siRNA. Cells were treated with DMSO or bafilomycin A_1_. Protein levels were determined by Western blot (left panel). Autophagic flux was determined by subtracting LC3B-II levels under control conditions (ctr) from levels of bafilomycin A_1_ (Bafi) treated cells. (right panel) (n=3; mean+/SD; t-test: *p≤0.05, **p≤0.01).
